# Comprehensive analysis of long-term trends, meteorological influences, and ozone formation sensitivity in the Jakarta Greater Area

**DOI:** 10.1038/s41598-024-60374-2

**Published:** 2024-04-26

**Authors:** Sheila Dewi Ayu Kusumaningtyas, Kenichi Tonokura, Robi Muharsyah, Dodo Gunawan, Ardhasena Sopaheluwakan, Windy Iriana, Puji Lestari, Didin Agustian Permadi, R. Rahmawati, Nofi Azzah Rawaani Samputra

**Affiliations:** 1 Agency for Meteorology, Climatology, and Geophysics of the Republic of Indonesia (BMKG), Jl. Angkasa I, No.2, Kemayoran, Jakarta, 10720 Indonesia; 2 Department of Environment Systems, Graduate School of Frontier Sciences, The University of Tokyo, 5-1-5 Kashiwanoha, Kashiwa, Chiba 277-8563 Japan; 3 School of Meteorology, Climatology, and Geophysics (STMKG), Agency for Meteorology, Climatology, and Geophysics of Republic of Indonesia (BMKG), Pondok Betung, Tangerang Selatan, Indonesia; 4 Department of Environmental Engineering, Faculty of Civil and Environmental Engineering, Bandung Institute of Technology (ITB), Jl. Ganesa No. 10, Bandung, 40132 Indonesia; 5 Center for Environmental Studies, Bandung Institute of Technology (ITB), Jl. Sangkuriang No.42 A, Bandung, 40135 Indonesia; 6 Department of Environmental Engineering, Faculty of Civil Engineering and Planning, National Institute of Technology (ITENAS), Jl. PKH. Mustopha No.23, Bandung, 40124 Indonesia; 7 Jakarta Provincial Environmental Agency, Jl. Mandala V No.67, RT.1/RW.2, Cililitan, Jakarta, 13640 Indonesia

**Keywords:** Atmospheric chemistry, Atmospheric chemistry

## Abstract

Jakarta Greater Area (JGA) has encountered recurrent challenges of air pollution, notably, high ozone levels. We investigate the trends of surface ozone (O_3_) changes from the air quality monitoring stations and resolve the contribution of meteorological drivers in urban Jakarta (2010–2019) and rural Bogor sites (2017–2019) using stepwise Multi Linear Regression. During 10 years of measurement, 41% of 1-h O_3_ concentrations exceeded Indonesia’ s national threshold in Jakarta. In Bogor, 0.1% surpassed the threshold during 3 years of available data records. The monthly average of maximum daily 8-h average (MDA8) O_3_ anomalies exhibited a downward trend at Jakarta sites while increasing at the rural site of Bogor. Meteorological and anthropogenic drivers contribute 30% and 70%, respectively, to the interannual O_3_ anomalies in Jakarta. Ozone formation sensitivity with satellite demonstrates that a slight decrease in NO_2_ and an increase in HCHO contributed to declining O_3_ in Jakarta with 10 years average of HCHO to NO_2_ ratio (FNR) of 3.7. Conversely, O_3_ increases in rural areas with a higher FNR of 4.4, likely due to the contribution from the natural emission of O_3_ precursors and the influence of meteorological factors that magnify the concentration.

## Introduction

Surface ozone (O_3_), a secondary pollutant from complex photochemical reactions between precursors and sunlight, adversely affects human health and vegetation^1,2^. Changes in photochemical activity and meteorological parameters can alter surface O_3_ concentrations^3–5^. Elevated temperatures and solar radiation accelerate the production of O_3_^6^. Stagnant conditions and lower planetary boundary layers have also been linked with high O_3_^7^_._ Surface O_3_ formation is a nonlinear process involving nitrogen oxides (NO_x_) and volatile organic compounds (VOCs) as precursors^8,9^. Thus, precursor emission changes can also modulate O_3_ concentration in addition to meteorological factors. NO_x_ is primarily produced from anthropogenic sources, whereas VOCs arise from both natural (biogenic) and anthropogenic sources. O_3_ sensitivity to the precursors depends on the photochemical regime of the O_3_ formation^10^. Photochemical O_3_ formation is influenced by a ratio of VOC to NO_x_. The formation regimes are generally classified into VOC-limited regime (where O_3_ formation is sensitive to an increase in VOCs), NO_x_-limited regime (where O_3_ concentration is mainly affected by an increase in NO_x_ and is insensitive to VOCs), and can be in a transitional state^4,11–13^. Understanding O_3_ formation sensitivity is essential for establishing practical O_3_ abatement efforts and remains a scientific challenge that must be addressed. Formaldehyde (HCHO) to nitrogen dioxide (NO_2_) Ratio (FNR), derived from satellite measurements, serves as a valuable indicator for inferring O_3_ formation due to its brief lifespan in the boundary layer and close association with O_3_ formation^14–16^. Martin et al.^14^ and Duncan et al.^15^ combined satellite measurements and air quality models to establish the FNR threshold and concluded that FNR < 1 indicates a VOC-limited regime, FNR > 2 indicates a NOx-limited regime, and 1 > FNR > 2 indicates a transitional regime in the United States. Jin et al.^17^ recently derived the FNR threshold for major U.S. cities with higher FNR values (3–4) in the transitional regime. In addition, Wang et al.^18^ revealed that a VOC-limited regime occurs when FNR < 2.3, a NO_x_-limited regime when FNR > 4.2, and an FNR between 2.3 and 4.2 reflects the transition regime in major Chinese cities.

Extensive research using modeling, ground measurements, and satellites has delved into surface O_3_ trends and causes, and its formation sensitivity, yet Southeast Asia, particularly Indonesia, remains inadequately explored, as evidenced by limited studies^19–21^. The scarcity of O_3_ measurements and a predominant focus on particulate matter reduction in Indonesia likely contribute to this research gap^22^. Indonesia currently installs 56 Air Quality Monitoring Stations (AQMS), twelve located in the Jakarta Greater Area (JGA). According to Verma et al.^23^, considering the vast population of 273.8 million, the number of AQMS in Indonesia is still very few and requires 1039 more stations. JGA is Indonesia’ s largest urban agglomeration, comprising Jakarta’ s capital and satellite cities such as Bogor, Depok, Bekasi, and Tangerang. The JGA has a dense population, rapid economic development, intensive industrialization, and high transportation utility. Bogor, located in the southern part of Jakarta, encompasses an area five times larger than that of Jakarta. With distinctive topography, Bogor functions as a downwind location influenced by local and transported emissions. An air quality station in Cibeureum (CBR), southeast Bogor, reflects a background environment close to Jakarta’ s.

This article unveils the seasonal variations and temporal trends in O_3_ concentrations within urban Jakarta and rural Bogor employing ozone metrics. The investigation assesses the contribution of meteorological and anthropogenic factors to O_3_ changes utilizing stepwise Multiple Linear Regression (MLR). Furthermore, the study analyzes O_3_ formation sensitivity using FNR derived from satellite observations and compares them with the previous studies. This study extends beyond Jakarta, offering insights with broader implications for other megalopolitan areas grappling with similar environmental challenges.

## Results

### Seasonal variation and temporal trends of ozone (O_3_)

**Figure 1:**
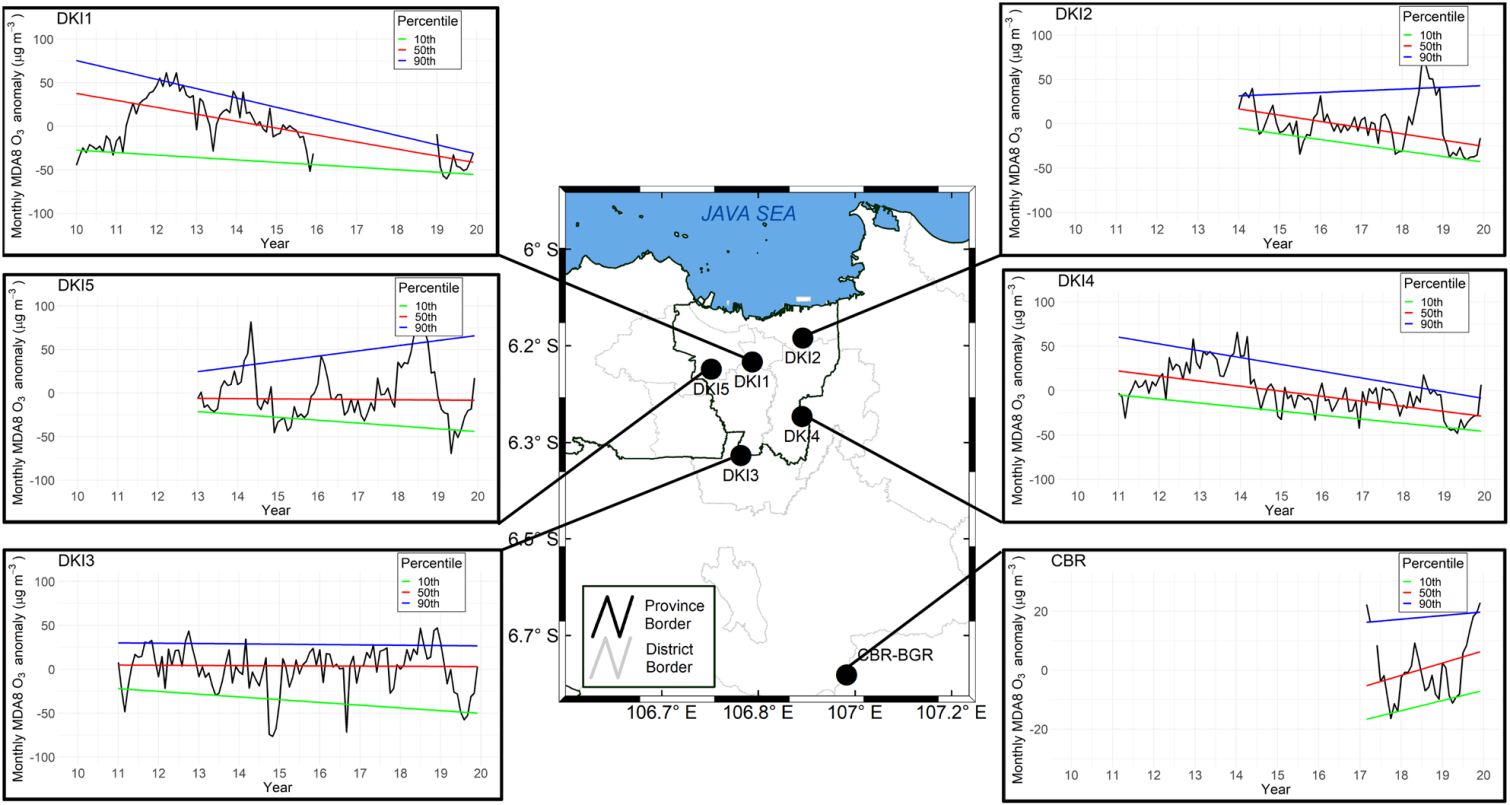
Map of the study area and the time series of the monthly mean anomaly of ozone maximum daily 8-h average (MDA8 O_3_) during the study period at Jakarta and Bogor sites. Color solid lines denote linear trends in the 90th (blue), 50th (red), and 10th (green) percentiles. Pink shade represents Jakarta Province with sites in central Jakarta (DKI1), north (DKI2), south (DKI3), east (DKI4), and west (DKI5). CBR represents the site in rural Bogor. The shapefile for Jakarta Greater Area boundary map is obtained from Geospatial Information Agency of Indonesia https://geoservices.big.go.id/petarbi/ and the map plot is generated using ArcGIS Pro online 2.8.0. The plot graph is generated using RStudio 1.4.1106. 2021.

Figure 1 depicts the time series of maximum daily 8-h average (MDA8) monthly anomalies and the trends in the 10th, 50th, and 90th percentiles at five sites in urban Jakarta (hereinafter refers to DKI) and one rural site of CBR. All DKI sites generally exhibit a downward trend of surface O_3_ in the 50th percentile, as presented in Table 1. Highest O_3_ declining rate occurs in DKI1, DKI2, DKI4, DKI5, and DKI3 by about − 8.0 µg m^−3^ year^−1^, − 7.1 µg m^−3^ year^−1^, − 5.7 µg m^−3^ year^−1^, − 0.3 µg m^−3^ year^−1^, and − 0.2 µg m^−3^ year^−1^, respectively. The tenth percentile represents low O_3_ concentration that may not involve or is less involved in photochemical reaction^24^. DKI2 in northern Jakarta appeared to have the highest and significant declining rate of − 6.4 m^−3^ year^−1^ for the 10th percentile, followed by DKI4 (− 4.6 µg m^−3^ year^−1^), DKI5 (− 3.3 µg m^−3^ year^−1^), DKI3 (− 3.1 µg m^−3^ year^−1^), and DKI1 (− 2.8 µg m^−3^ year^−1^).

**Table 1:** Slope values from QR annual trend analysis for different percentiles at DKI (2010–2019) and CBR sites (2017–2019).

Site	90th percentile	50th percentile	10th percentile
DKI1	**− 10.7 (*p* = 0.00072)**	− 8.0	− 2.8
DKI2	1.9	− 7.1	**− 6.4 (*p* = 0.00017)**
DKI3	− 0.4	− 0.2	− 3.1
DKI4	**− 7.7 (*p* = 0.000015)**	**− 5.7 (*p* = 0.0038)**	**− 4.6 (*p* = 0.00012)**
DKI5	5.9	− 0.3	− 3.3
CBR_BGR	1.3	4.2	3.4

Bold indicates 95% significance two-tailed test reached.

Meanwhile, MDA8 O_3_ anomaly trends in the 90th percentile show different directions from one site to another. Central (DKI1), south (DKI3), and east (DKI4) Jakarta shows a decreasing trend in different order of magnitude. DKI1 and DKI4 experience faster concentration decreases and significant rates of − 10.7 µg m^−3^ year^−1^ and − 7.7 µg m^−3^ year^−1^. DKI1 and DKI4 perform at faster-decreasing rates than their lower (50th and 10th) percentiles, indicating reduced extreme ozone episodes during the study period. Despite the reduction rate of O_3_ in the 50th and 10th percentiles, DKI2 and DKI5 present insignificant upward trends in the 90th percentile with a rate of 1.9 µg m^−3^ year^−1^ and 5.9 µg m^−3^ year^−1^, respectively, suggesting increase number of extreme O_3_ concentration.

The MDA8 O_3_ (average from all DKI sites) exceedance frequency (> 100 µg m^−3^ for 8-h threshold) in the dry season showed a sharp rise after 2010, fluctuated until 2018, but underwent a remarkable reduction in 2019, as demonstrated in Supplementary Fig. S1a. The minimum (maximum) exceedance frequency was observed in 2019 (2012). A yearly average of MDA8 O_3_ concentration surpassing the 8-h threshold, calculated from daily data, shows a slight reduction from 2011 to 2019 (see Supplementary Fig. S1b). This finding corresponds with the significant downward trend in high O_3_ concentration (90th percentile) observed at specific DKI sites, as depicted in Fig. 1 and Table 1.

**Figure 2:**
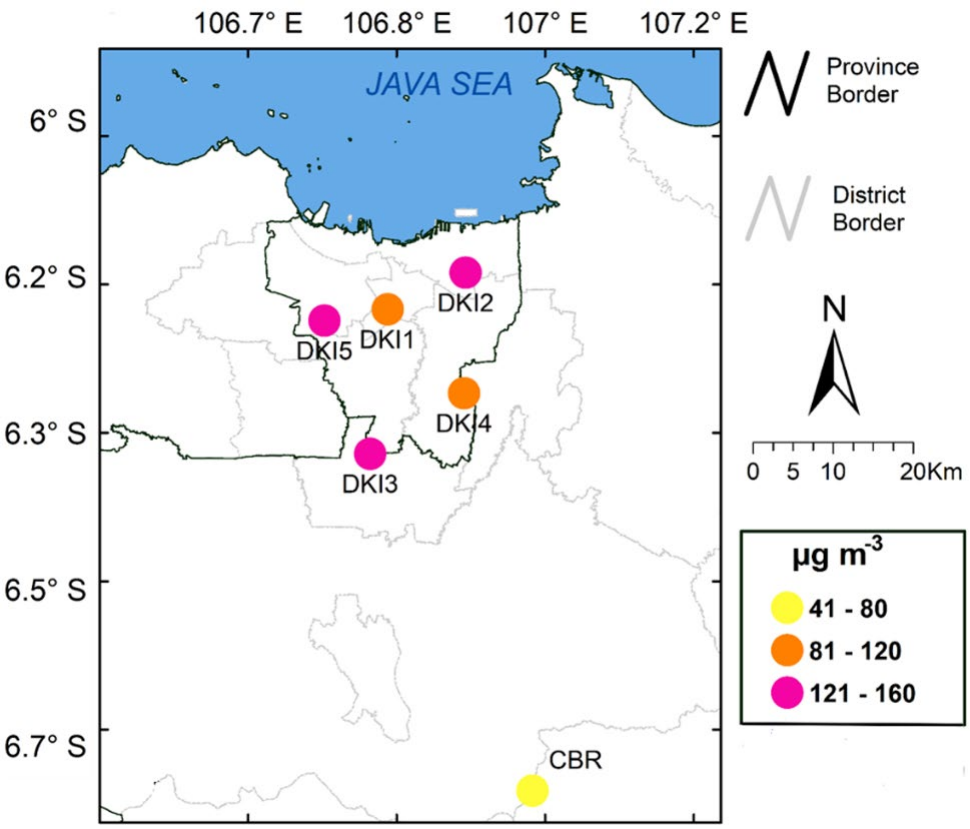
Map of the average of Maximum Daily 8-h ozone (AVGMDA8) in urban (Jakarta) and rural (Bogor) during the dry season (April to November) over the entire study period. (ArcGIS Online Pro 2.8.0. 2021). The shapefile for Jakarta Greater Area boundary map is obtained from Geospatial Information Agency of Indonesia https://geoservices.big.go.id/petarbi/ and the map plot is generated using ArcGIS Pro online 2.8.0.

The MDA8 O_3_ increased during the dry period across DKI and CBR sites, peaking in October. Figure 2 displays a map of the average maximum daily 8-h of ozone (AVGMDA8) in urban Jakarta and rural Bogor during the study period for the dry season (April to November). The AVGMDA8 varied and showed consistency with the 1-h O_3_ value, with the highest concentration at DKI3, followed by DKI5, DKI2, DKI4, and DKI1 with values of 128, 127, 122, 111, and 99 µg m^−3^, respectively. During 10-year period from 2010 to 2019, all DKI sites exceeded the National Ambient Air Quality Standard (NAAQS) for 1-h concentration of O_3_ (1-h O_3_ >150 µg m^−3^) with 18%, 26%, 30%, 37%, and 41% of exceedances, respectively, at DKI1, DKI4, DKI2, DKI3, and DKI5, as presented in Supplementary Fig. S2. The CBR experienced the 1-h O_3_ concentration threshold exceedances of 0.1% during 2017 to 2019. The monthly variations in 1-h O_3_ at the DKI and CBR sites peaked between May and November with an upward trend. The O_3_ concentrations gradually decreased from December to April. During the observation period, DKI5 experienced the highest annual average of 1-h O_3_ concentration compared to other sites with a maximum (minimum) of 194 µg m^−3^ (97 µg m^−3^), followed by DKI2 and DKI3, which were 160 µg m^−3^ (107 µg m^−3^) and 147 µg m^−3^ (103 µg m^−3^), respectively.

The overall O_3_ level at the CBR site was considered low compared to that in DKI sites and the NAAQS; however, the annual 1-h and MDA8 values increased from 2017 to 2019. MDA8 O_3_ anomaly increased in all percentiles by approximately 1.3 µg m^−3^ year^−1^ (90th), 4.2 µg m^−3^ year^−1^ (50th), and 3.4 µg m^−3^ year^−1^ (10th), indicating the enhanced presence of precursors that have critical roles in photochemical O_3_ formation in this area. Changing O_3_ concentration in the 10th percentile represents changes in the local background of O_3_ concentration^24^.

### Meteorological impact on the MDA8 O_3_ variation

**Figure 3:**
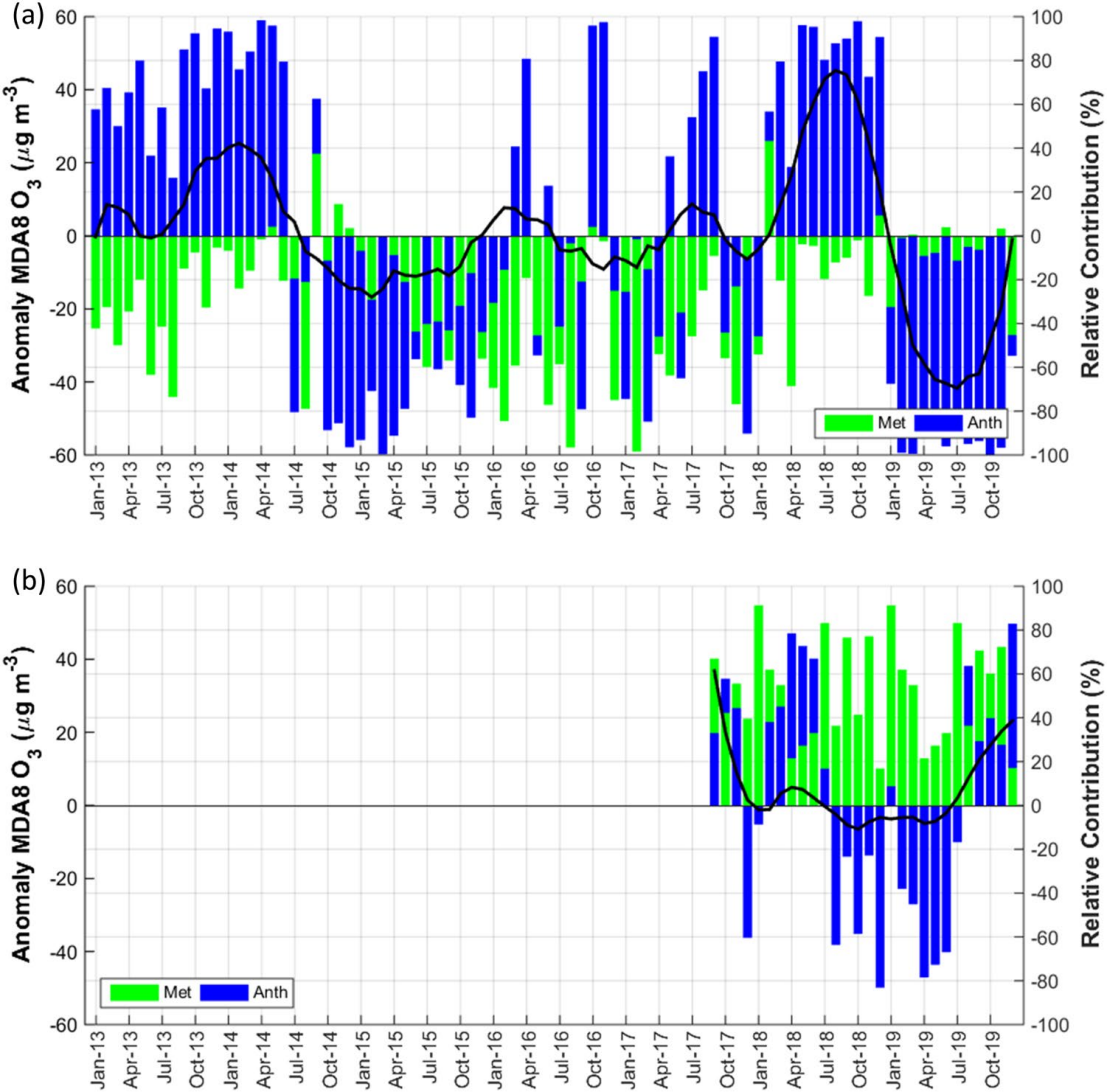
Contribution of meteorological (green bar) and anthropogenic factor (blue bar) to observed MDA8 O_3_ anomalies (black line) in (**a**) Jakarta (2013–2019) and (**b**) Bogor (2017–2019). (Matlab R2014b, 2014. 416,517).

The MLR model yielded the adjusted coefficients of determination (*R*^2^) for Jakarta (average from five DKI sites) and CBR areas for all seasons of 0.28 and 0.43, respectively, implying that 28% and 43% of the MDA8 O_3_ daily variability were associated with meteorological conditions. The contributions of meteorological and anthropogenic factors to the monthly mean MDA8 O_3_ anomalies in Jakarta and CBR areas over the entire period are depicted in Fig. 3a,b, respectively. Meteorological components influenced inter-annual O_3_ changes in Jakarta, accounting for 30% of the relative contribution for the entire season of 2013–2019. In general, meteorological components contribution ranged from 0.2 to 98.5% and was not considerably different from that of the dry season (Supplementary Fig. S3a). A slightly lesser meteorological influence shows during the wet season with a 25% relative contribution to O_3_ variability.

Pronounced positive contributions from meteorological features such as February 2018 altered MDA8_obs_ very slightly. Anthropogenic factors contributed 70% to the inter-annual variability of ΔMDA8_obs_, averaging 1.5–99.8%. Notably, positive ΔMDA8_obs_ in 2013, half of 2014, and 2018 were primarily attributed to anthropogenic rather than meteorological. The positive anthropogenic contribution reached more than 90% and was responsible for the increase of monthly mean ΔMDA8_obs_ during those years. The monthly mean of ΔMDA8_obs_ fluctuated and increased significantly in 2018 (Fig. 3a). These results indicate that changes in anthropogenic emissions are more favorable for O_3_ production, particularly during the dry season.

Downward UV radiation at the surface (UVB) significantly contributed to the O_3_ variation in all seasons, although the coefficient was minimal (Table 2). UVB also revealed a positive correlation with the correlation coefficient of *r* = 0.32, the second-highest value after wind speed (Supplementary Table S2). The UVB pattern was synchronous with maximum temperature (Tmax), particularly during the dry season, as shown in Supplementary Fig. S4c,d. Intense UV radiation is favorable for ozone production. Furthermore, higher temperatures can increase HCHO concentrations from biogenic emissions^25^. More than 70% of the daily observed MDA8 was above the NAAQS and coincided with UVB > 70,000 J m^−2^. According to Indonesian Meteorological Office (BMKG), the UV index in Jakarta at 10:00 local time was estimated to be 2–6, indicating a moderate to high risk. A negative anomaly of UVB influenced the O_3_ decline in 2013 (− 8.5 µg m^−3^) (Supplementary Fig. S4d). However, the high negative anomaly of UVB in 2016 (− 7759 J m^−2^) was likely canceled out by the positive contribution of Relative Humidity (RH), thus inducing only a small perturbation in O_3_.

**Table 2:** Coefficient of several variables from the multiple linear regression (MLR) model results for Jakarta (2013–2019) and CBR sites (2017–2019) in the dry, wet, and all seasons.

	Variable	Jakarta (2013–2019)			CBR (2017–2019)		
		All seasons	Dry season	Wet season	All seasons	Dry season	Wet season
Selected variable	WS_MEAN	− 22	− 24.72	− 4.98	− 5.93	3.98	
	UVB	0.00034	0.000514	0.00051	0.00023	0.00037	0.0003278
	TMAX	4.77	2.71	3.60	1.66	− 1.41	1.18
	RH	− 0.695	− 0.372	− 0.954	0.36		0.713
	BLH	− 0.010	− 0.016	− 0.0908	0.082	0.064	− 0.0404
	SP	− 0.0149	− 0.01	− 0.0378	0.0238	0.00125	
Intercept		1516	948	3700	− 2329	− 1156	− 0.74
*R*^2^		0.28	0.20	0.30	0.43	0.47	0.16

*P* value coefficient is presented in Supplementary Table S3.

UVB and wind speed mean (WS_MEAN) showed higher correlations than other parameters in Jakarta. The wind speed significantly (*p* = 2 × 10^−16^) contributed to the stepwise MLR. Wind speed is negatively correlated with MDA8 O_3_. During the dry period, air stagnation frequently occurred in Jakarta, boundary layer height (BLH) was low, calm winds dominated and recorded as approximately 70% of prevailing winds, and the wind speed was < 3 m s^−1^ leading to pollutant accumulation on the surface^26,27^. There is likely a contribution from the significant negative anomalies of the mean wind speed in 2018 (− 0.2 m s^−1^) to the increased MDA8_met_ (5.9 µg m^−3^) during the dry period (in Supplementary Fig. S3a). The BLH contributed negatively to Jakarta’ s MDA8 O_3_ change, indicating that shallow BLH increases O_3_ concentration. In addition, the immense positive contribution from emission drivers in 2018 substantially influenced the overall increase in O_3_ levels at the DKI sites. High RH is associated with enhanced cloud cover and a greater chance of rainfall, slowing the photochemical reaction of ozone formation^3,4^.

Meteorological and anthropogenic components shared almost equal proportions over 3 years’  period of measurement from 2017 to 2019 at CBR site, with average proportions of 52% (ranging from 17 to 91%) and 48% (ranging from 9 to 83%), respectively. However, the number did not change significantly during the dry season. UVB (*p* = 3 × 10^−6^), BLH (*p* < 2 × 10^−16^), Tmax (*p* = 0.005), and Surface Pressure (SP) (*p* = 1.7 ×10^−6^) significantly contributed to O_3_ variation at CBR. The CBR site exhibits complex terrain and thus presents unique weather conditions. High-altitude areas experience lower pressure. With a forced inversion layer and slow wind speeds, vertical mixing and horizontal dispersion in the atmosphere are poor and hindered by mountains, leading to increased concentrations of pollutants^28^. BLH was significant in CBR and had moderate positive correlations. In higher-altitude areas, during the development of a boundary layer, intrusion of O_3_ from the upper atmosphere to near the surface is possible, thus contributing to the surface O_3_ level^29,30^. This could be a possible reason for a positive correlation between BLH and O_3_ concentration. A similar characteristic of BLH and O_3_ variations observed at the CBR site is also evident in other locations with comparable geographic features. For instance, Mount Lulin in Taiwan^31^ and certain sites in in Beijing-Tianjin-Hebei Province and the Yangtze River Delta, China^4^.

### Spatial and temporal analysis of OMI NO_2_ and HCHO column density

The NO_2_ monthly average in Jakarta ranged from 1.5 to 8.5 × 10^15^ molecules cm^−2^, with a 10-year average of 3.6 × 10^15^ molecules cm^−2^ (Fig. 5a). This value is lower than those of cities in Japan^13^, China^9,11^, London^32^, and Mexico city^12^ for the same study period. The trend in the 50th percentile depicted an insignificant downward trend, with rates of − 0.04 × 10^15^ and − 0.01 × 10^15^ molecule cm^−2^ year^−1^ in Jakarta and CBR, respectively. The tropospheric NO_2_ in CBR is lower than in Jakarta, with a 10-year average of 2.9 × 10^15^ molecule cm^−2^ and ranging from 1.1 to 5.5 × 10^15^ molecule cm^−2^. Although the trend slightly decreased, a substantial increase was observed in the NO_2_ OMI from 2017 to 2019, with a slope of 0.02 × 10^15^ molecules cm^−2^ month^−1^.

**Figure 4:**
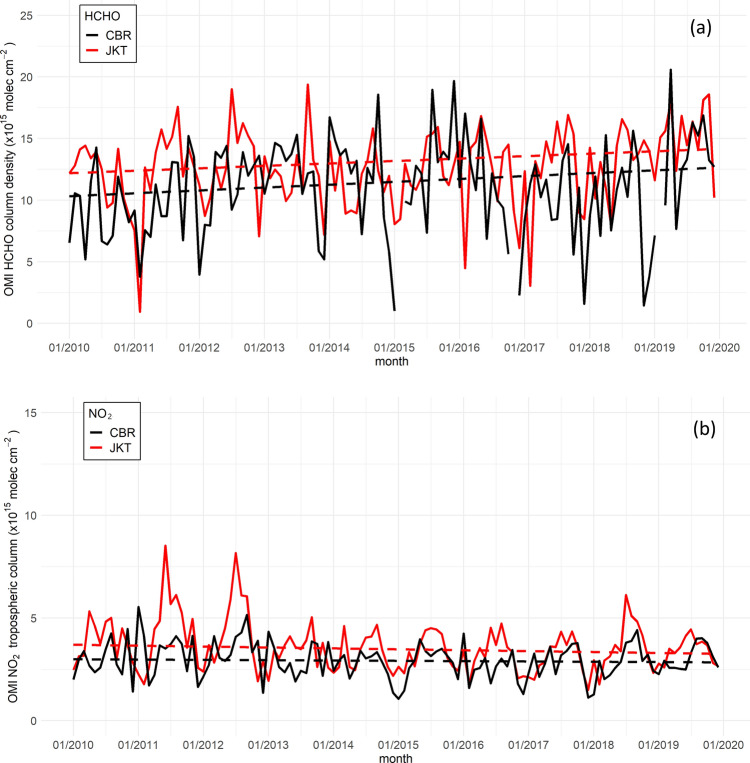
Monthly average of (**a**) HCHO column density and (**b**) NO_2_ tropospheric column from 2010–2019 in Jakarta and Cibeureum Bogor. The dashed line represents a trend in the 50th percentile. (RStudio 1.4.1106. 2021).

**Figure 5:**
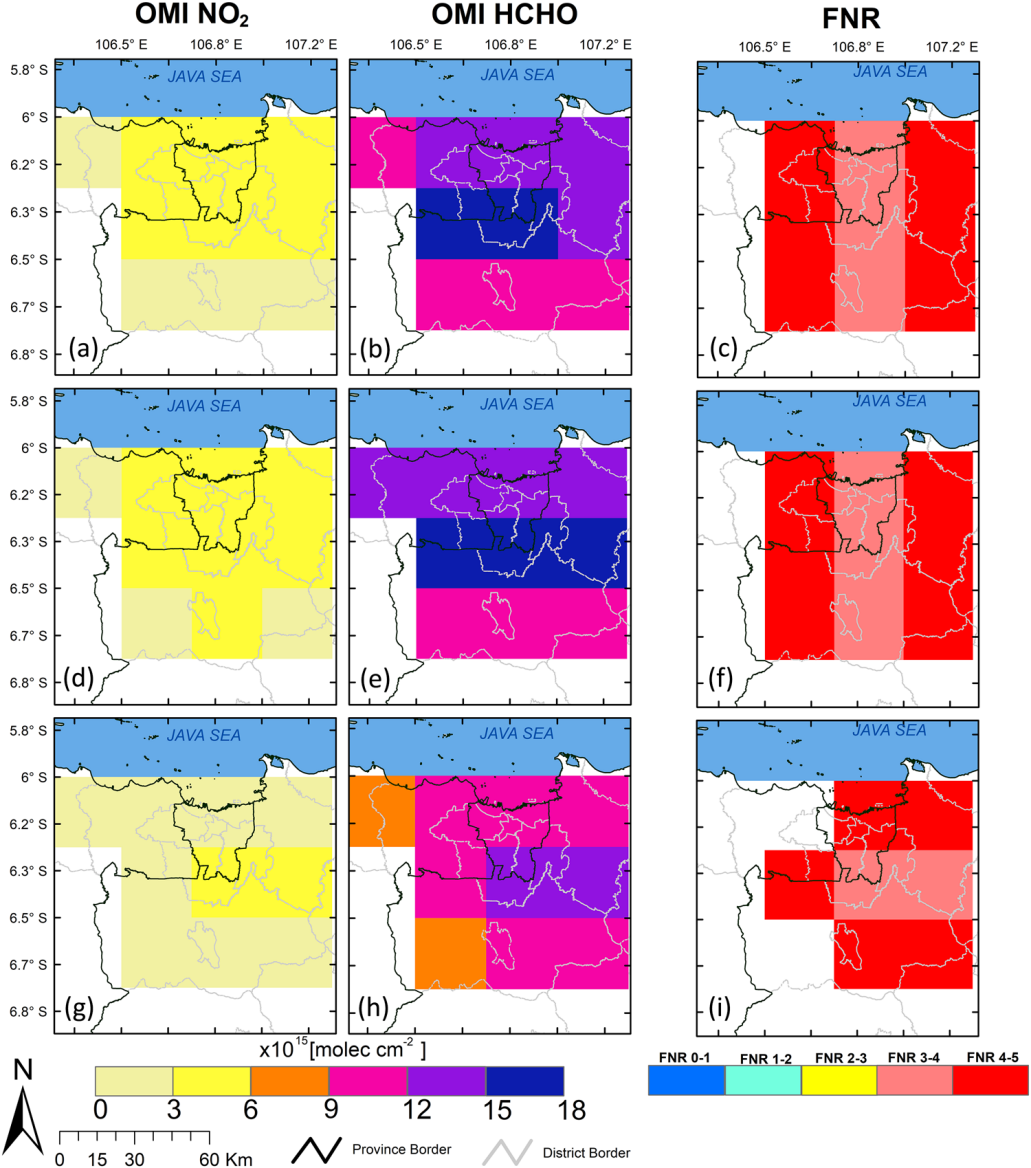
Map of the average (**a**) OMI NO_2_ tropospheric column, (**b**) OMI HCHO vertical column, and (**c**) FNR value from 2010–2019 for all seasons; (**d**), (**e**), and (**f**) for dry seasons; and (**g**), (**h**), and (**i**) for the wet season. Transparent areas indicate an FNR value above 5. The shapefile for Jakarta Greater Area boundary map is obtained from Geospatial Information Agency of Indonesia https://geoservices.big.go.id/petarbi/ and the map plot is generated using ArcGIS Pro online 2.8.0.

HCHO column density presented an insignificant upward trend of 0.19 × 10^15^ (0.23 × 10^15^) molecule cm^−2^ in Jakarta (CBR). 10-year average of OMI HCHO column density in urban Jakarta was 12.6 × 10^15^ molecules cm^−2^ with monthly average ranging from 0.9 to 19.4 × 10^15^ molecules cm^−2^ (Figs. 4a and 5b). This number is greater than that of urban areas in China (~2 to 18 × 10^15^ molecules cm^−2^ in Beijing and Shanghai), Japan (~1 to 16 × 10^15^ molecules cm^−2^ in Tokyo, Osaka, and Nagoya), and Mexico City (~3 to 9 × 10^15^ molecules cm^−2^)^12,33^.

OMI HCHO in CBR area is lower than in Jakarta, with a yearly average of 10.9 × 10^15^ molecules cm^−2^ over the entire study period (Fig. 5b). Biogenic VOC emissions from plants and vegetation are more reactive than anthropogenic VOCs, causing relatively high HCHO columns over cropland areas such as CBR. However, anthropogenic Non-Methane VOC (NMVOC) emissions surpass biogenic VOC emissions^11^ in urban cities where anthropogenic activities are massive and intense. Anthropogenic NMVOC emissions in Bogor from on-road motorized vehicles was around 9.7 Gg year^−1^ in 2016^34^, one-fifth smaller than Jakarta’ s emission for the same sector (48.6 Gg year^−1^ in 2015)^35^.

### Seasonal variation of OMI NO_2_ and HCHO

Seasonal variations in NO_2_ concentrations in Jakarta and CBR exhibited the same pattern, increasing in April and gradually decreasing in October. In Jakarta, the maximum (minimum) NO_2_ concentration occurred during the dry (wet) period in July (December) (Fig. 5d,g, and Supplementary Fig. S7). Enhanced column density occurred in northern Jakarta and extended to Bogor city center during the dry period (Fig. 5d). We also examine the monthly variability of NO_2_ from in-situ measurements and reveal that the concentration fluctuates throughout the year, and the seasonal pattern is not too obvious, as depicted in Supplementary Fig. S7. Sofyan et al.^36^ found no significant differences in fuel consumption in Jakarta between the dry and wet seasons. However, there is an apparent effect of sea breeze on transporting NO_2_ from north Jakarta to central and southern Jakarta. On dry days, converging sea breezes in the southern part of Jakarta cause pollutant accumulation in central Jakarta^36^. The pollutants then move along the sea breeze front and penetrate the Bogor area.

Generally, NO_2_ in Jakarta was higher than in CBR for the whole seasons. The maximum NO_2_ concentration in CBR occurred in September, and the minimum in December. The reduction in NO_2_ was mainly due to meteorological conditions during the wet period. Disregarding exceptional circumstances such as movement restrictions during the COVID-19 pandemic, which were not considered in this study, anthropogenic emissions in Jakarta and CBR were steady throughout the year.

Compared to NO_2_, HCHO was higher during the dry than wet period but fluctuated more (Fig. 5e,h). A slightly higher temperature during the dry period accelerated VOC photochemical oxidation, thereby contributing to high levels of HCHO. Elevated HCHO levels were notable and extended northwest and northeast of Jakarta during the dry period from 2010 to 2019, as shown in Fig. 5e. The HCHO column density in the whole Bogor area varied significantly seasonally. However, it was slightly lower in northwestern Bogor than in central Bogor.

### OMI FNR

Figure 5c presents the spatial yearly average FNR value from 2010 to 2019 in JGA. FNR in Jakarta steadily increased to 4.5, consistent with the increase in HCHO column density (Fig. 4a). Seasonal variations in the FNR occurred in Jakarta and CBR (Fig. 5f,i, and Supplementary Fig. S9b). As expected, the FNR decreased in Jakarta’ s dry period but fluctuated slightly in CBR. The reduction in FNR during the dry period was consistent with that shown in Supplementary Fig. S7, where the NO_2_ tropospheric column increased.

During the dry period, the maximum 1-h O_3_ and MDA8 O_3_ showed more days exceeding the 1-h and 8-h NAAQS thresholds than the wet period. It is worth noting that a large positive MDA8 O_3_ anomaly in 2018 was mainly driven by a large positive anomaly in anthropogenic activity. The increased MDA8 O_3_ is likely due to a response from the increase of OMI NO_2_ under a high FNR value of 3.9. However, to confirm this finding, further modeling work should be conducted.

## Discussion

O_3_ concentration and its formation mechanism are influenced by meteorology and changes in VOC and NO_x_. The O_3_ formation sensitivity could be either VOC-sensitive, NO_X_-sensitive, or transitional, where an increase of both precursors affects the O_3_ production. In a vibrant urban agglomeration such as JGA, characterized by intense emissions and limited and scattered AQMS, investigating the long-term trends of O_3_ pollutants and their formation regimes becomes imperative for effective air quality management. Identification of O_3_ formation sensitivity aids policymakers in designing appropriate control strategies. The current study evaluates trend and meteorological influences on O_3_ concentration variability. Additionally, we leverage space-borne NO_2_ and HCHO tropospheric column density to compute FNR as a VOC/NO_X_ ratio proxy to characterize O_3_ formation regimes.

Generally, our results show a downward trend of MDA8 O_3_ anomalies at all sites except CBR of rural Bogor. Despite the reduction trend, some sites experienced an increasing trend of high O_3_ episodes, such as in northern and western Jakarta, and almost steady state in southern Jakarta. This feature leads to high episodes exceeding the national standard for 1-h and 8-h O_3_ concentration from 2010 to 2019. As much as 41% of 1-h O_3_ concentration exceeded the national threshold for the whole seasons from 2010 to 2019 and 93% for MDA8 O_3_ in the 2018 dry season for Jakarta, with annual averages reaching 158.9 µg m^−3^. It is worth noting that high positive anomalies of O_3_ during 2018 are influenced mainly by anthropogenic drivers rather than the meteorology, as can be seen in Fig. 3a. It is likely due to a response from the increase of OMI NO_2_ under increase FNR value of 3.9 (Fig. 4b). However, to confirm this finding, further modeling work should be conducted.

DKI1 is in the city center, while DKI2 is near the busy harbor, power plants, and industrial areas. Both sites are characterized by higher NO_X_ emissions owing to high anthropogenic activities, as reflected by higher NO_2_ concentration (see Supplementary Fig. S8). In the vicinity of high NO emissions, O_3_ is removed via NO titration. However, its concentration increases further downstream, as in DKI3 and DKI5. DKI3 and DKI5 presented higher exceedance episodes and the least declining O_3_ trend. During the dry period, wind direction toward Jakarta mainly originated from the east and southeast, allowing the transport of pollutants from outside Jakarta to the eastern part of Jakarta, where industrial and manufacturing centers are located (around the Bekasi industrial area), contributing to the increase in O_3_ concentration at DKI5 (western Jakarta). In July, the HYbrid Single-Particle Lagrangian Integrated Trajectory (HYSPLIT) model’ s backward trajectory showed that air mass transport originated from Karawang and passed through Bekasi and South Jakarta (Supplementary Fig. S10). Despite the synoptic scale of easterly and southeasterly winds during the dry season, Jakarta was affected by sea breezes. According to Kitada et al.^37^, the southerly land breeze carried pollutant-rich but ozone-poor air masses to the Jakarta coast at night. In the morning, photochemical reactions produce O_3_ in northern Jakarta, and sea breeze circulation develops in the lower layers under weak synoptic winds^37^. The sea breeze, opposite the easterly and southeasterly winds, became dominant owing to the easterly and southeasterly synoptic scales blocked below approximately 1 km by the mountains in the southern part of West Java. The O_3_-rich air mass was transported to southern Jakarta and Bogor as the sea breeze propagated inland up to 60 km from the Jakarta coastline^37,38^. This sea breeze phenomenon affects the convection activity and transport of atmospheric pollutants, which explains the high concentration of DKI3 downwind of Jakarta.

Measurement of OMI NO_2_ in Jakarta shows an insignificant decreasing trend and increasing HCHO from time to time. In metropolitan cities in the US, China, Mexico, and Japan, tropospheric NO_2_ outnumbers the HCHO. Thus, the O_3_ formation is VOC-limited regime, as shown in Table S5. However, that is not the case for Jakarta, where HCHO column leads. As a consequence, the FNR falls in higher value compared to those cities. Study from Lestari et al.^35^ shows that the most significant emission load was from CO, NO_X_, and NMVOC, at approximately 52.9, 143.9, and 48.6 kilotonnes in 2015, respectively. Significant contributors to NO_X_ were the road transportation sector (57%), power plants (24%), industrial sectors (15%), and residential areas (4%)^35,39,40^. Furthermore, by Lestari et al.^35^, the total emissions inventory of NMVOC in Jakarta was comparable to that of NO_X_ in the same sectors and years. Road transport was the major contributor to NMVOC in Jakarta, with a percentage of 96%, followed by industry at 2%, power plants and residential areas at 1%. According to the Ministry of Transportation of the Republic of Indonesia, there was a total transportation movement of 19.63 million movements/day in 2022. Road transport by motorcycles contributed to 88% of the total NMVOC emissions. Jakarta Provincial Agency documented that there were over 15.8 million motorcycles in the city in 2019, approximately a 2.7% increase from 2018. Motorcycles represent the dominant vehicle fleet in Jakarta Province, accounting for approximately 80% of the total vehicles, followed by cars (17%), trucks (3%), and buses (0.2%)^41^. Similar emission types were also found in Vietnam, where motorcycles mainly contributed to the VOC emissions in Hanoi and Ho Chi Minh City. Motorcycles comprise 80% of the total transportation in Vietnam, and the number of vehicles per 1000 population in Vietnam is lower than that in Jakarta^42,43^. Emissions from motorcycles are likely attributable to high HCHO column density in Jakarta. In addition, NMVOCs are abundant in certain areas, such as gas stations^44^ and solvent usage industries^45^.

Different features occurred at the rural CBR site. A 3-year measurement period shows that the O_3_ concentration is below the government threshold; however, it shows an increasing rate for all percentiles. The growing O_3_ concentration value at the rural site indicates the enhanced presence of precursors that have critical roles in photochemical O_3_ formation. Changes in O_3_ concentration in the 10th percentile represent changes in the local background of O_3_ concentration. Unlike Bogor, a decreasing trend in O_3_ was observed in the rural North China Plain, Europe, and North America, likely because of the implementation of the O_3_ control policy^24^.

Bogor has a lower population density and more vegetation than Jakarta. The number of vehicles in Bogor is two million, one-thirteenth of those in Jakarta (26 million)^41^. Bogor, as the outskirt of Jakarta, may receive pollutants transported from Jakarta along with the sea breeze; however, this may not contribute substantially to the total emissions of the CBR. Lower concentrations recorded at the CBR were expected owing to lower emission activity, local characteristics, and meteorological disparity.

Air pollution prevention policies have been implemented in Indonesia and locally in JGA for more than a decade. For example, national kerosene-to-LPG conversion for cooking has been proven to reduce emissions^40^. Decreasing particulate matter, sulfate, and nitrate concentrations in Jakarta since 2006 have been used as evidence for the successful implementation of national and local policies, such as Regulation No. 141 concerning the Utilization of Gas Fuel for Public Transportation and Local Government Operational Vehicles^46^. Another successful implementation policy was issuing the Ministry of Environment and Forestry decree number 20/2017 on the usage of EURO IV for gasoline vehicles in late 2018. One current policy is that of the Governor of the DKI Jakarta, Regulation No. 66/2020 on vehicle emission testing, which regulates penalties for vehicles that violate the regulations.

Despite its ongoing implementation of measures, O_3_ pollution episodes persist. A more aggressive NO_X_ reduction should be in place in Jakarta. Thorough investigations of the precursors and emission inventory calculation are essential to enhance our understanding of O_3_ formation. Moreover, the thresholds for regime classification in Jakarta may require adjustment compared to global FNR thresholds. Further researches, combining modeling and observations, are warranted to identify effective O_3_ reduction scenarios for informed policy recommendations.

## Summary and conclusion

This study investigated variations and temporal trends in surface O_3_ under two different background conditions: urban (Jakarta, 2010–2019) and rural (Bogor, 2017–2019). We assessed the contributions of meteorological and anthropogenic factors and the long-term O_3_ formation sensitivity derived from OMI measurements. Despite the downward trend of O_3_ anomalies attributed to a slight reduction of NO_2_ concentration, Jakarta experiences recurrent elevated O_3_ level surpassing both the 1-h and 8-h national thresholds. Generally, anthropogenic drivers are more dominant than meteorological, irrespective of seasonal variations. However, O_3_ pollution can be even more severe under changing climate. Further research will focus in modeling simulations to validate the O_3_ sensitivity findings presented in this paper and eventually to establish O_3_ chemical regime thresholds, which are crucial for devising effective strategies to mitigate O_3_ pollution in JGA.

## Data description and methodology

### Study sites and air quality data

Jakarta, the center of government, business, and economic activities, contributes the most to the national gross domestic product with over 190 million USD in 2019^47^. We used air quality data from five AQMS in Jakarta (2010–2019) operated by the Environmental Agency of Jakarta Province, one station in Cibeureum Bogor (2017–2019) operated by the Meteorological, Climatological, and Geophysical Agency (BMKG) in collaboration with the National Institute of Environmental Studies (NIES), Japan, as presented in Fig. 1 and Supplementary Table S1. Further local characteristics of Jakarta and Bogor are described in Supplementary Table S6. We used hourly O_3_ concentrations and preprocessed the data to eliminate negative values and outliers. The time series of the hourly concentrations were normalized using the z-score following the methodology used by Ren et al. and Sun et al.^24,48^. Points with an absolute z-score greater than 4 (|zt|> 4) were removed from the time series. The daily average was calculated when a station had more than 60% hourly data, and we considered daily data missing if the availability was below this threshold.

### Surface O_3_ measurement

Jakarta Environmental Agency continuously measures 30-min surface O_3_ using HORIBA APOA-370 analyzer. The APOA-370 continuously monitors atmospheric ozone concentrations using a cross flow modulated ultraviolet absorption method or Non-dispersive ultraviolet absorption (NDUV). The NDUV measures ozone O_3_ concentration in sample gases using UV light absorption. UV light is emitted into a gas cell where the sample gas flows, and O_3_ absorbs UV light proportionally to its concentration. The absorbed light is detected by a photodiode, generating electrical signals. NDUV employs a cross-modulation method with a solenoid valve unit to switch between sample and reference gases, allowing the detector to distinguish AC and DC signals during processing. AC signals represent gas concentration, while DC signals compensate for UV light source aging, ensuring analyzer stability. CBR site in collaboration with NIES measure O_3_ concentration continuously using Kimoto OA-787^49^.

### Meteorological data

Meteorological data were used to construct the stepwise MLR model. Parameters such as Tmax, rainfall (RRR), WS_mean, and RH of Jakarta and Bogor were obtained from the BMKG. Other meteorological parameters, such as the BLH, SP, and UVB, were downloaded from the European Center for Medium-Range Weather Forecasts Reanalysis (ECMWF ERA5) product on single levels with a spatial resolution of 0.25° × 0.25° and a temporal resolution of 1 h for the corresponding grid, similar to the meteorological stations and AQMS (https://cds.climate.copernicus.eu/cdsapp#;/dataset/).

### Satellite product

We use level three gridded data of the daily ozone-monitoring instrument (OMI)^50^ NO_2_ tropospheric column standard product (OMNO2d_003) with a cloud fraction of less than 30% and a spatial resolution of 0.25° × 0.25° downloaded from https://disc.gsfc.nasa.gov/. To calculate the ratio, we average the NO_2_ daily data to monthly. The monthly level three vertical column density of OMI HCHO was downloaded from the European Quality Assurance for Essential Climate Variables project (QA4ECV) (http://www.qa4ecv.eu) with a spatial resolution of 0.05° × 0.05°. The QA4ECV HCHO slant column densities (SCDs) have 8–12 × 10^15^ molecule cm^−2^ uncertainties. We re-grid the monthly OMI HCHO data to 0.25° × 0.25° to match the monthly OMI NO_2_ data.

### Trend analysis of ozone metrics

To identify O_3_ pollution, we use an indicator from the Indonesian Government Regulation No. 22 Year 2021 on the implementation of environmental protection and management for 1-h O_3_ concentration (measured from 11:00 to 14:00 local time) and O_3_ metric following the Tropospheric Ozone Assessment Report (TOAR), such as the maximum daily 8-h average (MDA8)^51^. This metric is intended to evaluate the impact of O_3_ on human health, vegetation, model comparisons, and the characterization of O_3_ in the free troposphere^51^. MDA8 O_3_ was calculated from 24 running 8-h averages. The 8-h running mean for a particular hour is computed based on the concentration for that hour plus the following 7 h. The daily maximum 8-h concentration for a given calendar day was the highest of the 8-h average concentrations computed for 8-h periods starting from that day. The 8-h running mean was considered valid when at least 5 h of data were available (60% completeness was required). The average MDA8 (AVGMDA8) is the mean value of daily MDA8 (calculated based on more than 60% of the daily MDA8).

Quantile Regression (QR) estimates the trend in MDA8 O_3_ concentration. QR is a well-suited technique for detecting heterogeneous distributional changes, which is often the case for free tropospheric and surface ozone as they typically show diverse percentile trends^52^. Trend uncertainty is estimated by doing a moving block bootstrap algorithm to take autocorrelation into account in the trend uncertainty. Trend analysis considers the seasonality of MDA8 O_3_ time series by calculating the MDA8 O_3_ anomaly. MDA8 monthly anomalies are more accurate than the monthly average in case of missing data^10,48,53^. Monthly anomalies were calculated by subtracting the individual monthly mean from the monthly mean for the same month for the entire study period. Meanwhile, zero anomaly refers to a situation where the monthly MDA8 O_3_ is equal to the long-term average value or no deviation or departure from the average conditions. QR analysis is computed using Rstudio with a quantreg package.

### Stepwise multiple linear regression (MLR) model

Statistical models were used to infer the contributions of meteorological and anthropogenic factors to the monthly ozone variability. Researchers commonly use the MLR to quantify the effects of meteorological factors on O_3_^4,6,9,10,24^. The MLR uses the following equation to reflect the relationship between a quantitative dependent variable and two or more independent variables:

1 $${{\varvec{y}}}_{{\varvec{s}},\boldsymbol{ }{\varvec{c}}}={{\varvec{\beta}}}_{{\varvec{o}}}+{\sum }_{{\varvec{k}}=1}^{{\varvec{n}}}{{\varvec{\beta}}}_{{\varvec{s}},\boldsymbol{ }\boldsymbol{ }{\varvec{c}}}\boldsymbol{ }{\varvec{x}}\boldsymbol{ }{{\varvec{M}}{\varvec{e}}{\varvec{t}}}_{{\varvec{k}}}+\boldsymbol{ }{\varvec{\varepsilon}}$$

The stepwise MLR estimated MDA8 O_3_ from meteorological factors (MDA8_met_); where *y* is the daily observed MDA8 O_3_ (MDA8_obs_) in season *s* (dry, wet, and all seasons) and city *c* (Jakarta and Bogor); Met_k_ is the selected daily meteorological parameter; β_s,c_ is the regression coefficient in season *s* and city* c*; β_o_ is the intercept; and ɛ is the residual. MDA8_obs_ used to construct the model were averaged from the MDA8_obs_ at five sites in Jakarta and one for rural Bogor.

A series of steps was conducted to determine the relative meteorology contribution to O_3_ variation. First, correlation coefficients were established between MDA8_obs_ and all meteorological parameters (see Supplementary Table S2). Second, the Variance Inflation Factor (VIF) was calculated to avoid multicollinearity between variables. Variables with a VIF > 10 indicated multicollinearity removal, and those with a 10 were retained. In this study, all the predictors had a VIF < 10 (approximately 1–3) and were retained for the next selection step. Third, stepwise regression was conducted to obtain the best model fit by adding or removing predictors based on Akaike Information Criterion (AIC) statistics. In this step, optimal meteorological parameters were selected and used to establish an MLR model. The optimal meteorological parameters, calculated intercept (*β*_o_), regression coefficient (*β*_*s,c*_), and adjusted R-squared for Jakarta (2013–2019) and Bogor (2017–2019) in the dry, wet, and all seasons are listed in Table 2, and Supplementary Table S3, S4. The MDA8 O_3_ resulting from the stepwise MLR model shows the contribution of the meteorological component (MDA8_met_). The difference between MDA8_obs_ and MDA8_met_ is called the residual and is considered an anthropogenic driver attributed to the ozone variability (MDA8_ant_)^9,10,29^.

The total change in meteorological contribution (ΔMDA8_met_) to O_3_ concentration can be calculated as follows:

2 $${{\varvec{\Delta}}\mathbf{M}\mathbf{D}\mathbf{A}8}_{\mathbf{m}\mathbf{e}\mathbf{t}}={\sum }_{{\varvec{k}}=1}^{{\varvec{n}}}{{\varvec{\beta}}}_{{\varvec{s}},{\varvec{c}}}\boldsymbol{ }{\varvec{x}}\boldsymbol{ }{{\varvec{\Delta}}\mathbf{M}\mathbf{e}\mathbf{t}}_{\mathbf{k}}$$

where ΔMet_k_ is the change in the k-th meteorological variable. The change in MDA8_ant_ (ΔMDA8_ant_) was the difference between the change in observed MDA8 (ΔMDA8_obs_) and ΔMDA8_met_. Thus, the relative contribution of meteorology to the ozone concentration could be calculated as the ratio of ΔMDA8_met_ to the total meteorological and anthropogenic factors (ΔMDA8_met_ + ΔMDA8_ant_).

### Ozone formation sensitivity (OFS)

A common approach involves using satellite measurements to determine observed VOC-to-NO_X_ ratios (FNR). FNRs have been widely used to study OFS. The tropospheric NO_2_ column reflects NO_X_ emissions at the surface because most NO_2_ satellite measurements are within the planetary boundary layer and quickly transform or are removed from the atmosphere (short lifetime)^14,18^. HCHO is an intermediate product of all VOCs, and can be used as a proxy for VOC reactivity^54^. HCHO is a short-lived molecule that is usually found near emission sources.

The FNR threshold for separating the VOC-sensitive regime from the NO_x_-sensitive regime was derived using various methods for urban areas in the U.S. and China. The FNR threshold can vary by location owing to the influence of precursor emissions, meteorology, and geography^17,55^. This study compared FNR values in Jakarta and Bogor with those reported in the previous studies. The flowchart and methodology used in this study are shown in Supplementary Fig. S11.

### Supplementary Information

Supplementary Information.

## Data Availability

This study used the products of OMI NO_2_ and HCHO from NASA (USA) and European Commission Project for Quality Assurance for Essential Climate Variables (QA4ECV). Meteorological data are available from BMKG (Indonesia) and ECMWF ERA5. Ground O_3_ and NO_2_ data belong to Jakarta Environmental Agency.
