# Supplementary material for: Comprehensive analysis of long-term trends, meteorological influences, and ozone formation sensitivity in the Jakarta Greater Area
Source: Sci Rep. 2024 Apr 26;14:9605. doi: 10.1038/s41598-024-60374-2 (PMC11053138; doi:10.1038/s41598-024-60374-2)
Supplement: Supplementary file 1 — Supplementary Information. [file 41598_2024_60374_MOESM1_ESM.docx]

# Comprehensive Analysis of Long-Term Trends, Meteorological Influences, and Ozone Formation Sensitivity in the Jakarta Greater Area

# Sheila Dewi Ayu Kusumaningtyas1,2*, Kenichi Tonokura^2^*, Robi Muharsyah^1^, Dodo Gunawan^3^, Ardhasena Sopaheluwakan^1^, Windy Iriana^4,5^, Puji Lestari^4^, Didin Agustian Permadi^6^, Rahmawati^7^, & Nofi Azzah Rawaani Samputra^7^

1Agency for Meteorology, Climatology, and Geophysics of the Republic of Indonesia (BMKG), Jl. Angkasa I, No.2, Kemayoran, Jakarta 10720, Indonesia

2Department of Environment Systems, Graduate School of Frontier Sciences, The University of Tokyo, 5-1-5 Kashiwanoha, Kashiwa, Chiba 277-8563, Japan

^3^School of Meteorology, Climatology, and Geophysics (STMKG), Agency for Meteorology, Climatology, and Geophysics of Republic of Indonesia (BMKG), Pondok Betung, Tangerang Selatan, Indonesia

^4^Department of Environmental Engineering, Faculty of Civil and Environmental Engineering, Bandung Institute of Technology (ITB), Jl. Ganesa No. 10, Bandung 40132, Indonesia

^5^Center for Environmental Studies, Bandung Institute of Technology (ITB), Jl. Sangkuriang No.42 A, Bandung 40135, Indonesia

^6^Department of Environmental Engineering, Faculty of Civil Engineering and Planning, National Institute of Technology (ITENAS), Jl. PKH. Mustopha No.23, Bandung 40124, Indonesia

^7^Jakarta Provincial Environmental Agency, Jl. Mandala V No.67, RT.1/RW.2, Cililitan, Jakarta 13640, Indonesia

*corresponding:sheila.dewi@bmkg.go.id

*corresponding: tonokura@k.u-tokyo.ac.jp

Supplementary:

Table S1. Air quality and meteorological data used in this study.

Table S2. Pearson correlation coefficients (𝑟) between daily MDA8 ozone and meteorological variables in Jakarta (2013 to 2019) and CBR sites (2017 to 2019). Bold indicates positive or negative correlation coefficients of more than 0.3.

Table S3. P-value of predictors variables coefficient from the multiple linear regression (MLR) model results for Jakarta (2013–2019) and CBR sites (2017–2019) in the dry, wet, and all seasons.

Table S4. Uncertainty of relative contribution (%) from meteorological drivers in Jakarta and CBR.

Table S5. Comparison of ozone formation sensitivity research results in other urban cities worldwide.

Table S6. Local characteristics of Jakarta and Bogor.

Figure S1. (a) Variation in frequent exceedances of the 8-h NAAQS threshold (100 µg/m^3^) of MDA8 O_3_ concentration (average from five DKI sites) and (b) yearly average exceeding concentration during the dry season (April-November).

Figure S2. Measurement of 1-h O_3_ concentration between 11:00 and 14:00 LT at five Jakarta sites of central DKI1 (a), north DKI2 (b), south DKI3 (c), east DKI4 (d), west DKI5 (e), and one rural Bogor CBR site (f). Red solid line indicates the National Ambient Air Quality Standard threshold for 1-h.

Figure S3. Contribution of meteorological (green bar) and anthropogenic factors (blue bar) to the observed MDA8 O_3_ anomalies during (a, c) the dry season (April to November) and (b, d) the wet season in DKI from 2013 to 2019 (top panel) and in CBR sites (bottom panel) from 2017 to 2019.

Figure S4. Temporal variations in MDA8 meteorology (blue solid line), (a) WS_mean, (b) RH, (c) Tmax, (d) UVB anomalies, (c) SP, and (f) BLH resulting from the MLR model in all seasons from 2013 to 2019 at the DKI sites.

Figure S5. Temporal variations in MDA8 meteorology (blue solid line), (a) WS_mean, (b) RH, (c) Tmax, (d) UVB anomalies, (c) SP, and (f) BLH resulting from the MLR model in the dry season from 2013 to 2019 at the DKI sites.

Figure S6. Temporal variations in MDA8 meteorology (blue solid line), (a) WS_mean, (b) RH, (c) Tmax, (d) UVB, (e) BLH, and (f) SP anomalies resulting from the MLR model during the wet season of 2013–2018 at the DKI sites.

Figure S7. Seasonal variation of OMI NO_2_ and HCHO column in Jakarta and CBR from 2010-2019.

Figure S8. Seasonal variation of ground observed NO_2_ concentration at DKI sites from 2010 to 2019.

Figure S9. Time series of the (a) annual average and (b) seasonal variation in FNR in Jakarta and CBR from 2010 to 2019.

Figure S10. HYSPLIT backward trajectory on July 7, 2017 (dry period) in Jakarta.

Figure S11. Flowchart and methodology used in this study.

Table S1. Air quality and meteorological data used in this study

| Station | Latitude, Longitude | Parameter | Elevation (m) | Background | Start date | End date | N (number of days) |
| --- | --- | --- | --- | --- | --- | --- | --- |
| DKI 1: Bundaran HI (Central Jakarta) | -6.194, 106.823 | O_3_ (1-h) | 3 | City center; roadside of the busy street; surrounded by building offices | Jan 2010 | Dec 2019 | 2436 |
| DKI 2: Kelapa Gading (North Jakarta) | -6.153, 106.910 | O_3_ (1-h) | 5 | Around the settlement; close to the harbor and Java Sea coast; industrial and shipping area | Jan 2014 | Dec 2019 | 2138 |
| DKI 3: Jagakarsa (South Jakarta) | -6.356, 106.803 | O_3_ (1-h) | 71 | Around the settlement and vegetation | Jan 2011 | Dec 2019 | 3148 |
| DKI 4: Lubang Buaya (East Jakarta) | -6.288, 106.909 | O_3_ (1-h) | 27 | Around the settlement and vegetation | Jan 2011 | Dec 2019 | 2997 |
| DKI 5: Kebon Jeruk (West Jakarta) | -6.207, 106.752 | O_3_ (1-h) | 6 | Around the settlement; instrument placed in the open field | Jan 2013 | Dec 2019 | 2402 |
| CBR:Cibeureum (South Bogor) | -6.735, 106.986 | O_3_ (1-h), maximum temperature (Tmax), rainfall (RRR), wind speed mean (WS_MEAN), relative humidity (RH), | 920 | Rural and mountainous area; close to the recreational spot and up in the hill; dense vegetation; 27 km south of the city center Bogor and 75 km from city center Jakarta | Jan 2017 | Dec 2019 | 930 |
| Kemayoran Meteorological Station (Central Jakarta) | -6.155, 106.840 | maximum temperature (Tmax), rainfall (RRR), wind speed mean (WS_MEAN), relative humidity (RH) | 4 | City center; roadside; surrounded by building offices | Jan 2013 | Dec 2019 | 2556 |
| Tanjung Priok Meteorological Maritime Station (North Jakarta) | -6.107, 106.880 |  | 3 | Close to the Jakarta harbor | Jan 2013 | Dec 2019 | 2556 |
| Soekarno Hatta Meteorological Station (West Jakarta) | -6.120, 106.650 |  | 11 | Inside the Jakarta airport neighborhood | Jan 2013 | Dec 2019 | 2556 |
| ECMWF ERA5 |  | Boundary Layer Heigh (BLH), UVB, SP |  |  | Jan 2013 | Dec 2019 | 2556 |

Table S2. Pearson correlation coefficients (𝑟) between daily MDA8 ozone and meteorological variables in Jakarta (2013 to 2019) and CBR sites (2017 to 2019). Bold indicates positive or negative correlation coefficients of more than 0.3.

| Met Parameter | DKI | | | CBR | | | |
| --- | --- | --- | --- | --- | --- | --- | --- |
|  | All seasons | Dry season | Wet season | All seasons | Dry season | Wet season |  |
| UVB | **0.32** | 0.23 | **0.35** | **0.50** | **0.53** | 0.27 |  |
| BLH | -0.024 | -0.01 | **-0.35** | **0.60** | **0.66** | -0.18 |  |
| SP | 0.01 | 0.02 | -0.06 | 0.27 | 0.28 | 0.13 |  |
| RH | -0.25 | -0.10 | 0.03 | **-0.46** | **-0.47** | 0.10 |  |
| TMAX | **0.30** | 0.14 | 0.21 | **0.40** | **0.30** | 0.14 |  |
| RRR | -0.15 | -0.05 | -0.04 | -0.21 | -0.23 | 0.02 |  |
| WS_MEAN | **-0.38** | **-0.35** | **-0.36** | 0.19 | **0.33** | -0.12 |  |

Table S3. P-value of predictors variables coefficient from the multiple linear regression (MLR) model results for Jakarta (2013–2019) and CBR sites (2017–2019) in the dry, wet, and all seasons. The star indicates the degree of significance. “***” indicates that the coefficient estimate is highly statistically significant at the 0.001 significance level. ”**” denotes statistical significance at the 0.01 significance level. “*” indicates statistical significance at the 0.05 significance level. “.” signifies that the coefficient estimate is marginally significant, with a p-value less than 0.1 but greater than or equal to 0.05. “ “ (blank) indicates that the coefficient estimate is not statistically significant at the conventional levels (p ≥ 0.1).

|  |  | Jakarta (2013-2019) | | | CBR (2017-2019) | | |
| --- | --- | --- | --- | --- | --- | --- | --- |
|  | Variable | All seasons | Dry season | Wet season | All seasons | Dry season | Wet season |
| Selected variable | WS_MEAN | < 2x10^-16^ *** | < 2e-16 *** | 0.003575 ** | 0.01503 * | 0.1696 |  |
|  | UVB | 6.15x10^-12^ *** | 3.75x10^-13^ *** | 6.19x10^-16^ *** | 2.91x10^-6^ *** | 2.87x10^-09^ *** | 3.95x10^-7^ *** |
|  | TMAX | 6.97x10^-13^ *** | 0.00198 ** | 0.000284 *** | 0.00502 ** | 0.0345 * | 0.15924 |
|  | RH | 0.000106 *** | 0.10156 | 0.000991 *** | 0.01458 * |  | 0.00492 ** |
|  | BLH | 0.209176 | 0.10862 | 5.88x10^-11^ *** | < 2x10^-16^ *** | < 2x10^-16^ *** | 0.00294 ** |
|  | SP | 0.003017 ** | 0.18863 | 9.49x10^-8^ *** | 1.72x10^-6^ *** | 0.0397 * |  |
| Intercept |  | 0.002704 ** | 0.16032 | 2.35x10^-7^ *** | 7.83x10^-7^ *** | 0.0445 * | 0.06165 |

Table S4. Uncertainty of relative contribution (%) from meteorological drivers in Jakarta and CBR.

| Month | **relative contribution of meteorology-driven change (%) for Jakarta** | | | **relative contribution of meteorology-driven change (%) for CBR** | | |
| --- | --- | --- | --- | --- | --- | --- |
|  | estimated value | lower 2.5% | upper 97.5% | estimated value | lower 2.5% | upper 97.5% |
| Jan-13 | -42.2 | -47.3 | -36.1 |  |  |  |
| Feb-13 | -32.5 | -42.8 | -17.8 |  |  |  |
| Mar-13 | -49.9 | -60.0 | -33.1 |  |  |  |
| Apr-13 | -34.5 | -40.0 | -27.9 |  |  |  |
| May-13 | -20.1 | -36.9 | 7.6 |  |  |  |
| Jun-13 | -63.4 | -82.0 | 52.4 |  |  |  |
| Jul-13 | -41.4 | -57.6 | -5.4 |  |  |  |
| Aug-13 | -73.6 | -90.1 | 78.1 |  |  |  |
| Sep-13 | -15.0 | -27.3 | 2.2 |  |  |  |
| Oct-13 | -7.6 | -15.6 | 2.0 |  |  |  |
| Nov-13 | -32.8 | -37.2 | -27.6 |  |  |  |
| Dec-13 | -5.4 | -12.2 | 2.5 |  |  |  |
| Jan-14 | -6.8 | -15.4 | 3.4 |  |  |  |
| Feb-14 | -24.0 | -35.4 | -7.9 |  |  |  |
| Mar-14 | -15.9 | -22.0 | -8.8 |  |  |  |
| Apr-14 | -1.6 | -13.2 | 10.7 |  |  |  |
| May-14 | -4.1 | -1.5 | 9.2 |  |  |  |
| Jun-14 | -20.5 | -37.9 | 8.7 |  |  |  |
| Jul-14 | -19.5 | -36.3 | 7.7 |  |  |  |
| Aug-14 | -79.0 | -83.6 | -70.7 |  |  |  |
| Sep-14 | -37.4 | -95.1 | 95.3 |  |  |  |
| Oct-14 | -11.3 | -30.3 | 15.2 |  |  |  |
| Nov-14 | 14.5 | -27.2 | 41.6 |  |  |  |
| Dec-14 | 3.5 | -7.0 | 13.0 |  |  |  |
| Jan-15 | -6.9 | -12.4 | -0.6 |  |  |  |
| Feb-15 | -29.2 | -45.0 | -0.4 |  |  |  |
| Mar-15 | -0.4 | -18.5 | 17.9 |  |  |  |
| Apr-15 | -8.8 | -18.7 | 3.6 |  |  |  |
| May-15 | -21.1 | -33.1 | -3.8 |  |  |  |
| Jun-15 | -43.7 | -59.1 | -9.6 |  |  |  |
| Jul-15 | -59.8 | -69.6 | -40.8 |  |  |  |
| Aug-15 | -39.1 | -48.8 | -24.8 |  |  |  |
| Sep-15 | -56.9 | -67.0 | -37.7 |  |  |  |
| Oct-15 | -31.9 | -52.8 | 15.2 |  |  |  |
| Nov-15 | -17.0 | -25.4 | -6.5 |  |  |  |
| Dec-15 | -56.1 | -62.2 | -47.6 |  |  |  |
| Jan-16 | -69.4 | -75.3 | -59.7 |  |  |  |
| Feb-16 | -84.5 | -91.7 | 15.1 |  |  |  |
| Mar-16 | -59.2 | -65.7 | -49.8 |  |  |  |
| Apr-16 | -19.2 | -33.4 | 2.5 |  |  |  |
| May-16 | -45.5 | -58.6 | -20.1 |  |  |  |
| Jun-16 | -77.1 | -86.1 | -34.8 |  |  |  |
| Jul-16 | -58.6 | -71.3 | -25.7 |  |  |  |
| Aug-16 | -96.5 | -97.8 | -91.5 |  |  |  |
| Sep-16 | -20.8 | -37.4 | 6.6 |  |  |  |
| Oct-16 | 4.0 | -77.0 | 77.4 |  |  |  |
| Nov-16 | -2.5 | -26.6 | 23.7 |  |  |  |
| Dec-16 | -74.9 | -78.8 | -69.1 |  |  |  |
| Jan-17 | -25.6 | -40.9 | 0.5 |  |  |  |
| Feb-17 | -98.5 | -99.1 | -93.5 |  |  |  |
| Mar-17 | -15.2 | -22.3 | -6.6 |  |  |  |
| Apr-17 | -54.0 | -60.9 | -44.2 |  |  |  |
| May-17 | -63.8 | -68.7 | -57.0 |  |  |  |
| Jun-17 | -35.0 | -52.0 | 0.3 |  |  |  |
| Jul-17 | -45.9 | -52.1 | -37.8 |  |  |  |
| Aug-17 | -24.9 | -37.5 | -6.1 |  |  |  |
| Sep-17 | -9.2 | -16.7 | -0.3 | 67.0 | 56.1 | 73.6 |
| Oct-17 | -55.9 | -69.4 | -20.5 | 42.2 | 26.2 | 52.5 |
| Nov-17 | -76.9 | -86.4 | -24.7 | 55.6 | 44.7 | 63.0 |
| Dec-17 | -9.7 | -19.3 | 2.3 | 39.6 | 28.6 | 47.6 |
| Jan-18 | -54.1 | -65.9 | -29.8 | 91.3 | 86.1 | 93.7 |
| Feb-18 | 43.3 | 19.1 | 56.4 | 61.9 | 43.0 | 71.4 |
| Mar-18 | -20.4 | -30.8 | -6.4 | 54.9 | 40.7 | 63.6 |
| Apr-18 | -68.6 | -76.8 | -51.0 | 21.5 | 8.7 | 31.1 |
| May-18 | -3.9 | -12.3 | 5.7 | 27.3 | 18.7 | 34.2 |
| Jun-18 | -4.6 | -8.8 | -0.1 | 33.0 | 25.0 | 39.5 |
| Jul-18 | -19.7 | -25.3 | -13.1 | 83.2 | 76.1 | 87.1 |
| Aug-18 | -12.2 | -17.3 | -6.4 | 36.3 | 25.7 | 44.3 |
| Sep-18 | -10.0 | -14.1 | -5.5 | 76.6 | 64.1 | 82.6 |
| Oct-18 | -2.1 | -5.2 | 1.2 | 41.4 | 33.4 | 47.6 |
| Nov-18 | -27.4 | -34.2 | -19.1 | 77.1 | 69.0 | 81.9 |
| Dec-18 | 9.2 | -7.9 | 22.4 | 16.9 | 9.6 | 23.0 |
| Jan-19 | -32.4 | -37.7 | -26.2 | 91.3 | 86.1 | 93.7 |
| Feb-19 | -1.0 | -12.7 | 11.2 | 61.9 | 43.0 | 71.4 |
| Mar-19 | 0.5 | -3.8 | 4.8 | 54.9 | 40.7 | 63.6 |
| Apr-19 | -9.2 | -11.3 | -7.0 | 21.5 | 8.7 | 31.1 |
| May-19 | -7.8 | -11.4 | -3.9 | 27.3 | 18.7 | 34.2 |
| Jun-19 | 3.9 | -6.0 | 12.7 | 33.0 | 25.0 | 39.5 |
| Jul-19 | -11.3 | -19.7 | -1.1 | 83.2 | 76.1 | 87.1 |
| Aug-19 | -5.0 | -11.4 | 2.3 | 36.3 | 25.7 | 44.3 |
| Sep-19 | -6.4 | -14.4 | 3.0 | 70.6 | 60.0 | 76.8 |
| Oct-19 | -0.2 | -9.5 | 9.3 | 60.2 | 47.8 | 67.8 |
| Nov-19 | 3.3 | -10.0 | 15.3 | 72.3 | 62.9 | 77.9 |
| Dec-19 | -45.2 | -52.9 | -34.4 | 17.1 | 9.2 | 23.7 |

Table S5. Comparison of ozone formation sensitivity research results in other urban cities worldwide.

| City (region) | Regime | Threshold | Method | Citation |
| --- | --- | --- | --- | --- |
| Los Angeles, New York City | VOC-limited | NO_X_ limited: >2 VOC limited: <1 | Modelling | ^1–4^ |
| LA, Chicago, NYC (2013–2016) | VOC-limited | NO_X_ limited: >4 VOC limited: <3 | Satellite and ground measurement |  |
| NCP, PRD China (2016–2019) | VOC-limited | NO_X_ limited: >4.2  VOC limited: <2.3 | Observation and modeling |  |
| PRD China (2014–2016) | VOC-limited to transitional regime (March and April) | NO_X_ limited: >2.39  VOC limited: <1.25 | Satellite and ground measurement  Statistical analyses |  |
| Mexico City | VOC-limited | NO_X_ limited: >2 VOC limited: <1 | Satellite and ground measurement | ^5^ |
| Tokyo (warm season 2015–2019) | VOC-limited | NO_X_ limited: >2 VOC limited: <1 | Satellite and ground measurement | ^6^ |

Table S6. Local characteristics of Jakarta and Bogor^7^

| Characteristics | Jakarta | Bogor |
| --- | --- | --- |
| Population | 10.6 million (census 2022) | 6 million (census 2022) |
| Topography | Flat with elevation 0 - 80 m asl. | Surrounded by two mountains, Salak and Gede Pangrango. Elevation ranging from 190 to 1200 m asl. |
| Total area | 664 km^2^ | 3104 km^2^ |
| Climate | Affected by the sea breeze circulation, with the northern part directly adjacent to the Java Sea.  Max temperature 32 °C  Average of total rainfall per year range from 1000 – 2000 mm  The dry (wet) season occurs from April to November (December to March) | Max temperature 26 °C  Average of total rainfall per year range from 2000 – 3000 mm |
| Local emission type | Transportation, industrial, power plant, residential | Agricultural burning, transportation, industrial, residential |


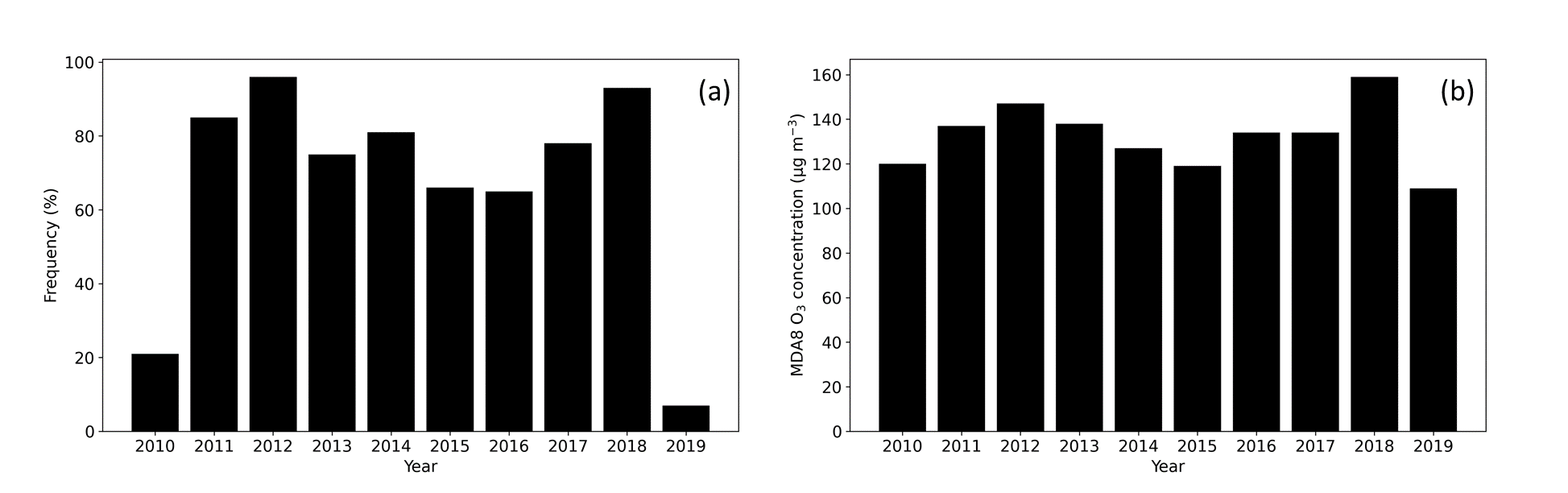


Figure S1. (a) Variation in frequent exceedances of the 8-h NAAQS threshold (100 µg/m^3^) of MDA8 O_3_ concentration (average from five DKI sites) and (b) yearly average exceeding concentration during the dry season (April-November).


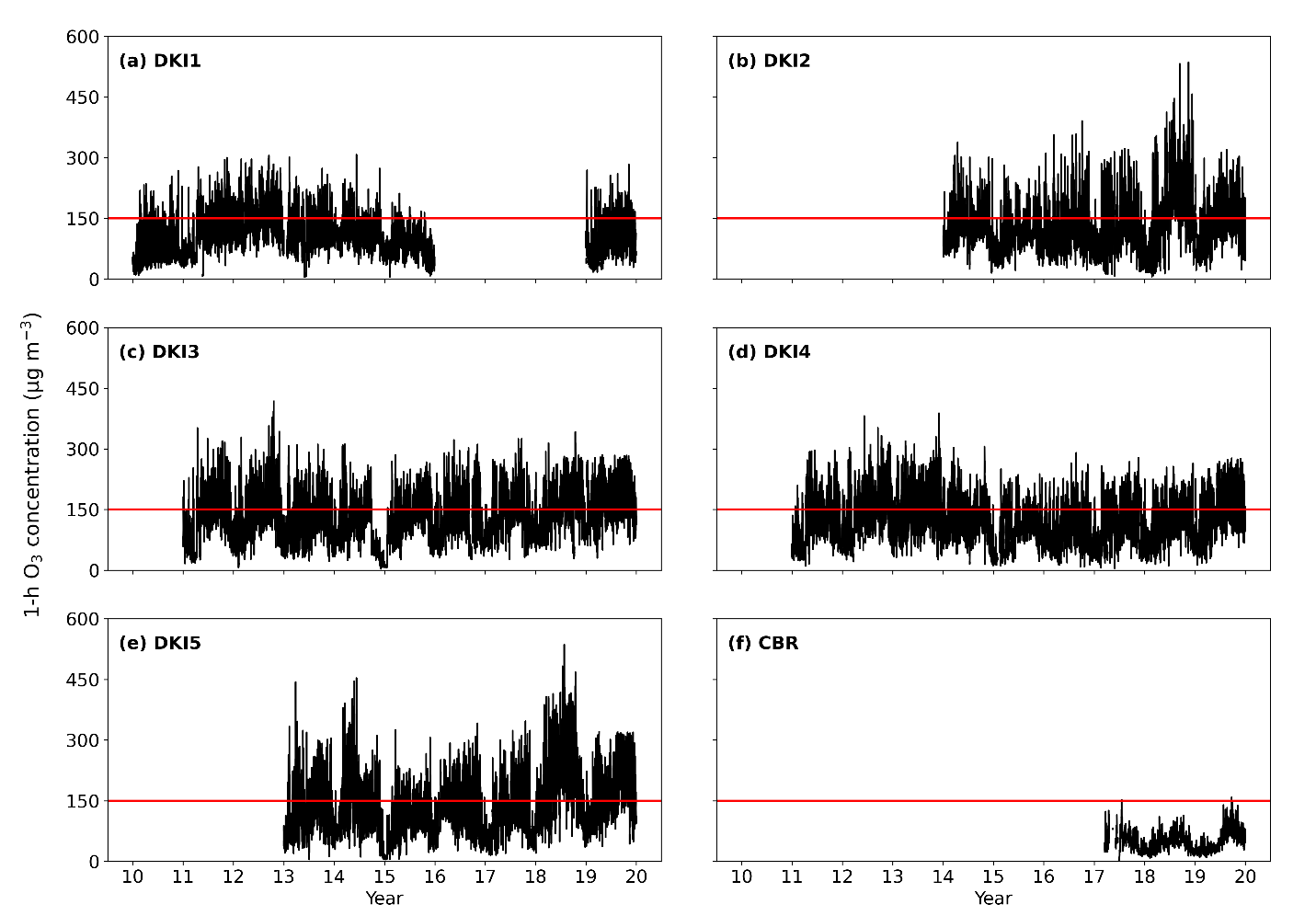


Figure S2. Measurement of 1-h O_3_ concentration between 11:00 and 14:00 LT at five Jakarta sites of central DKI1 (a), north DKI2 (b), south DKI3 (c), east DKI4 (d), west DKI5 (e), and one rural Bogor CBR site (f). Red solid line indicates the National Ambient Air Quality Standard threshold for 1-h.


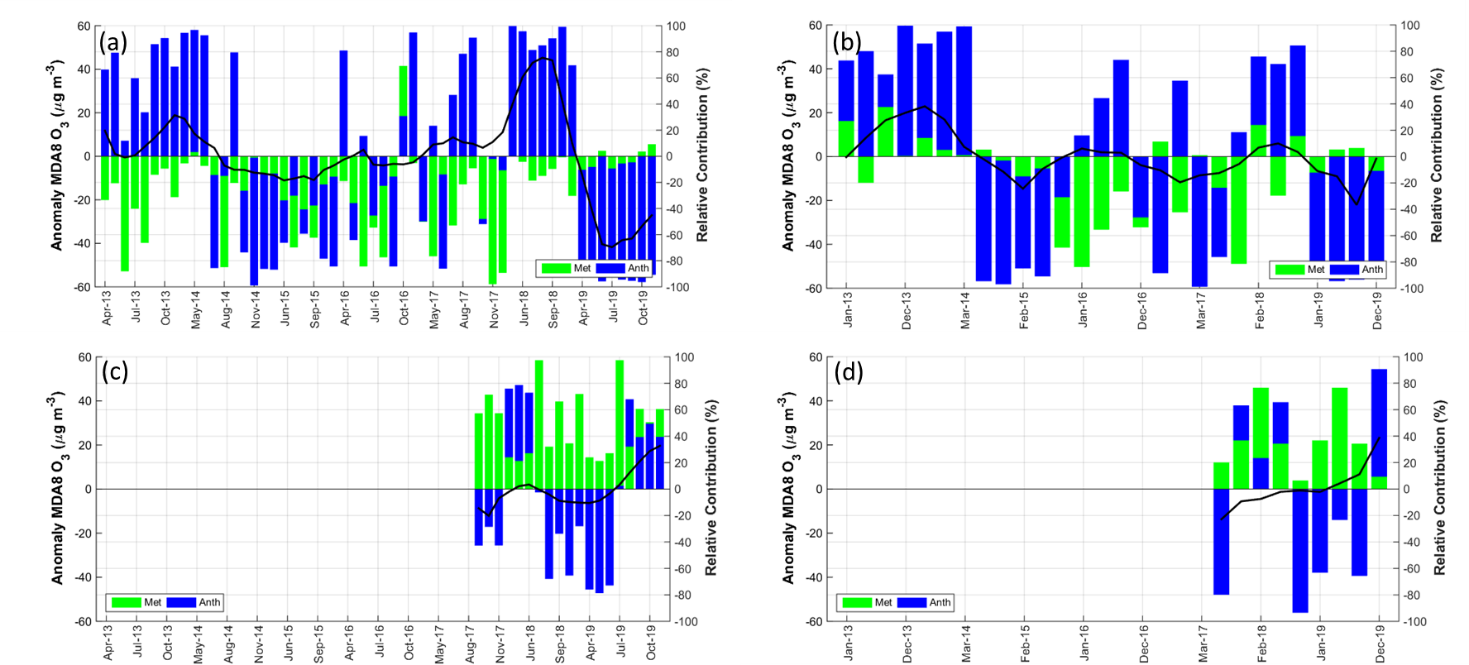


Figure S3. Contribution of meteorological (green bar) and anthropogenic factors (blue bar) to the observed MDA8 O_3_ anomalies during (a, c) the dry season (April to November) and (b, d) the wet season in DKI from 2013 to 2019 (top panel) and in CBR sites (bottom panel) from 2017 to 2019.


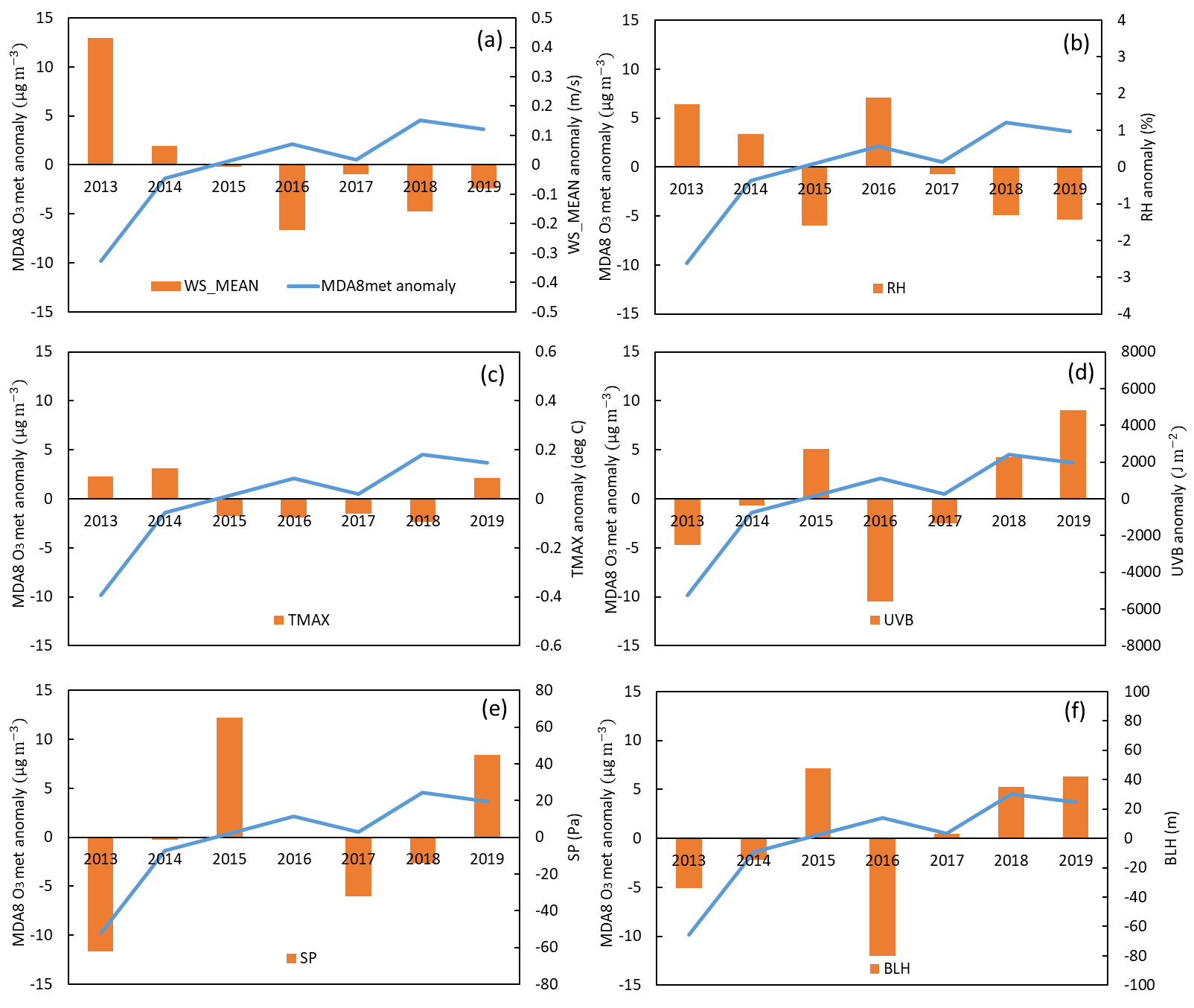


Figure S4. Temporal variations in MDA8 meteorology (blue solid line), (a) WS_mean, (b) RH, (c) Tmax, (d) UVB anomalies, (c) SP, and (f) BLH resulting from the MLR model in all seasons from 2013 to 2019 at the DKI sites.


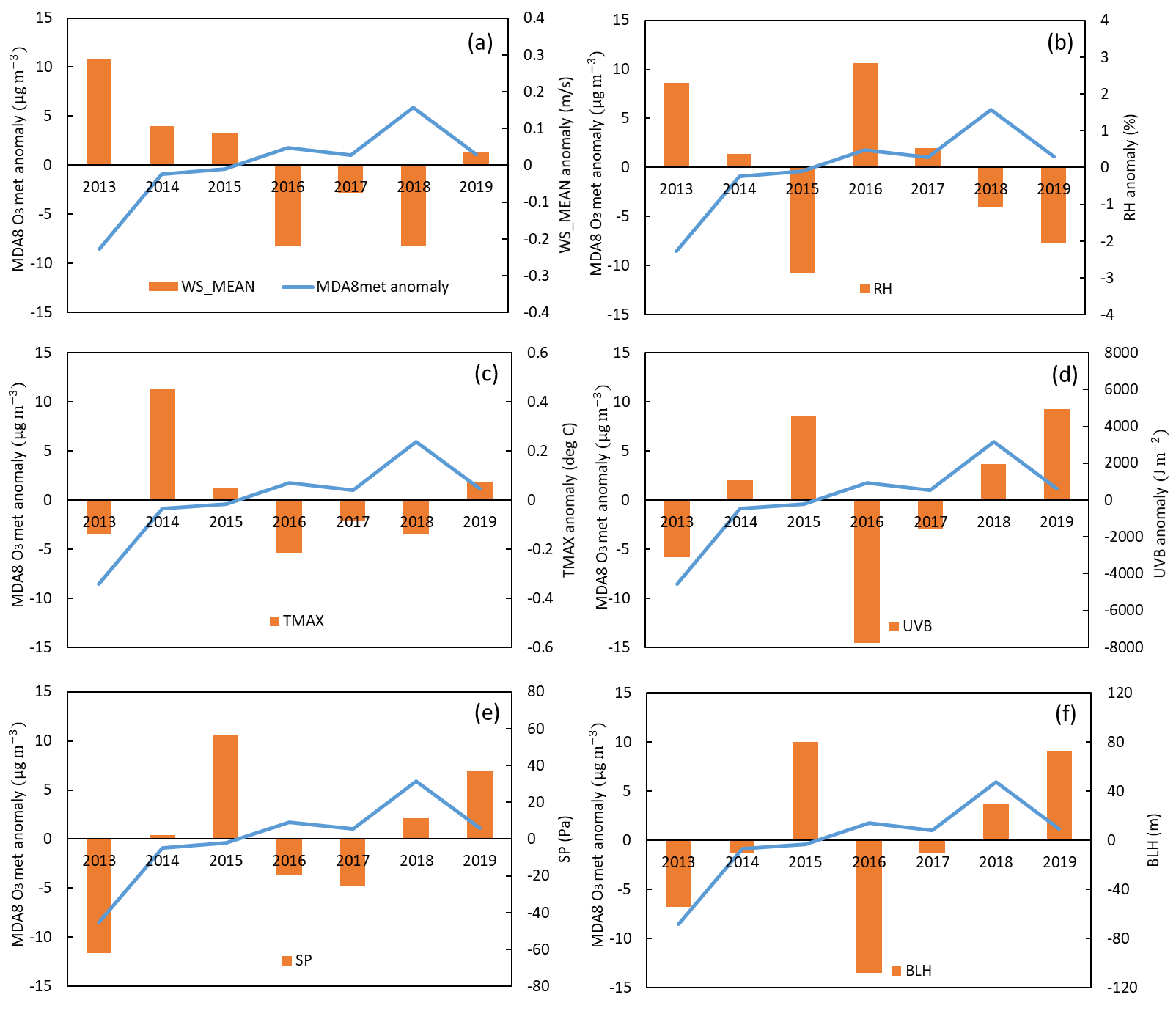


Figure S5. Temporal variations in MDA8 meteorology (blue solid line), (a) WS_mean, (b) RH, (c) Tmax, (d) UVB anomalies, (c) SP, and (f) BLH resulting from the MLR model in the dry season from 2013 to 2019 at the DKI sites.


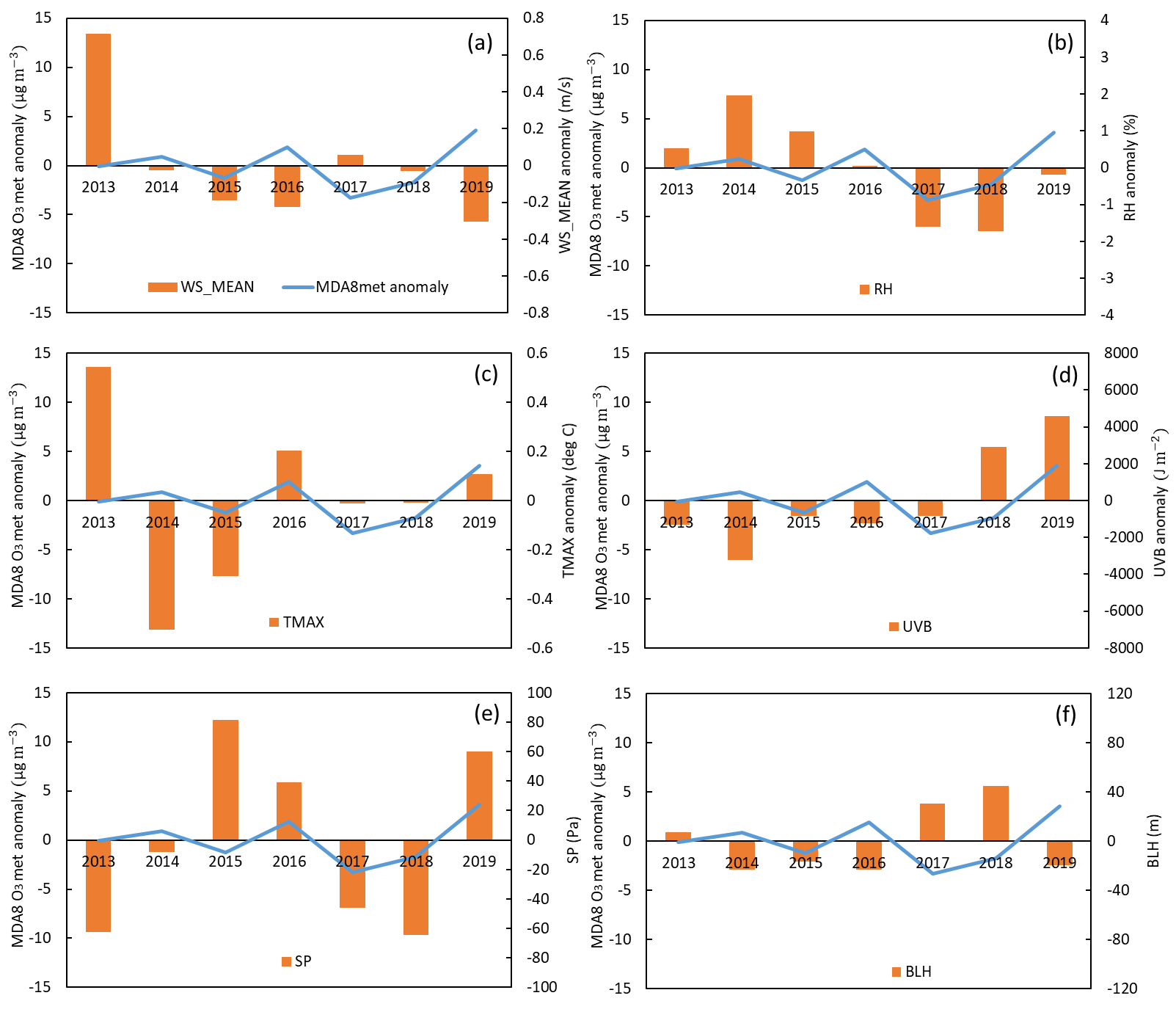


Figure S6. Temporal variations in MDA8 meteorology (blue solid line), (a) WS_mean, (b) RH, (c) Tmax, (d) UVB anomalies, (c) SP, and (f) BLH resulting from the MLR model in the wet season of 2013 to 2019 at the DKI sites.


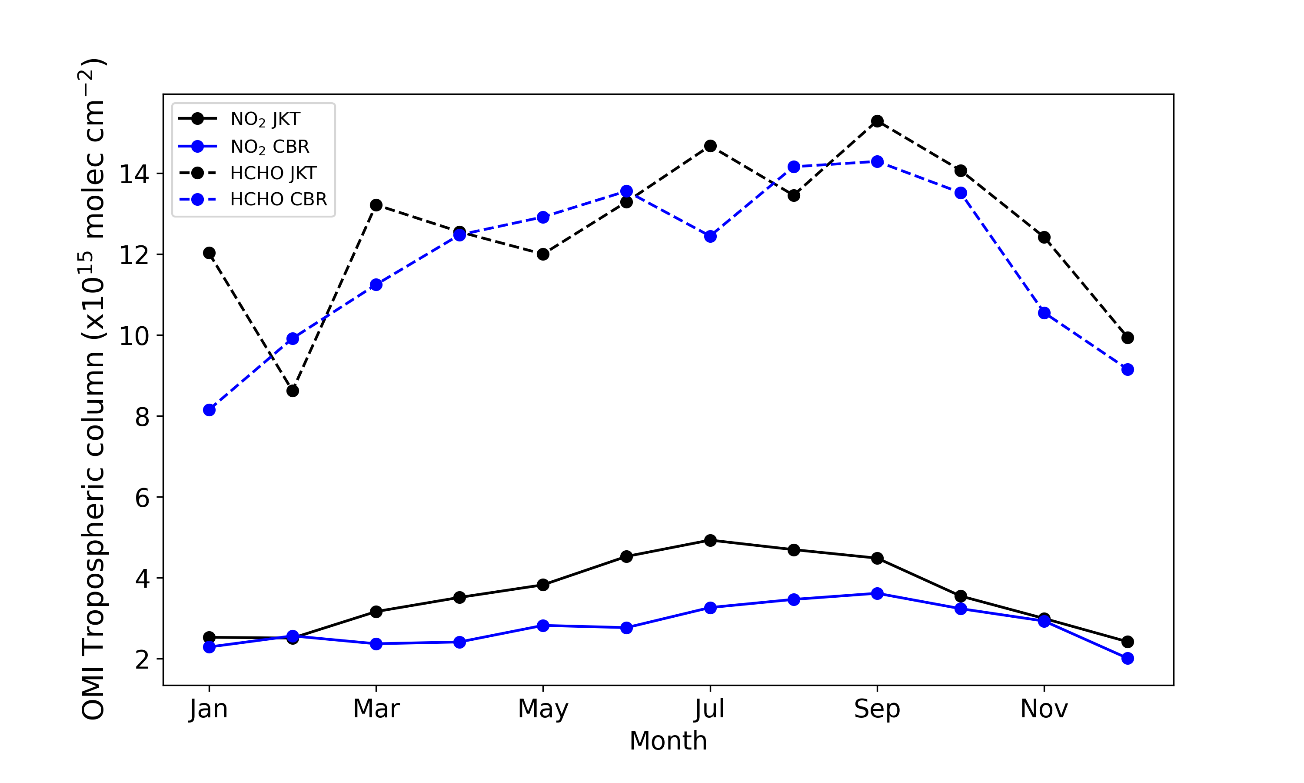


Figure S7. Seasonal variation of OMI NO_2_ and HCHO column in Jakarta and CBR from 2010 to 2019.


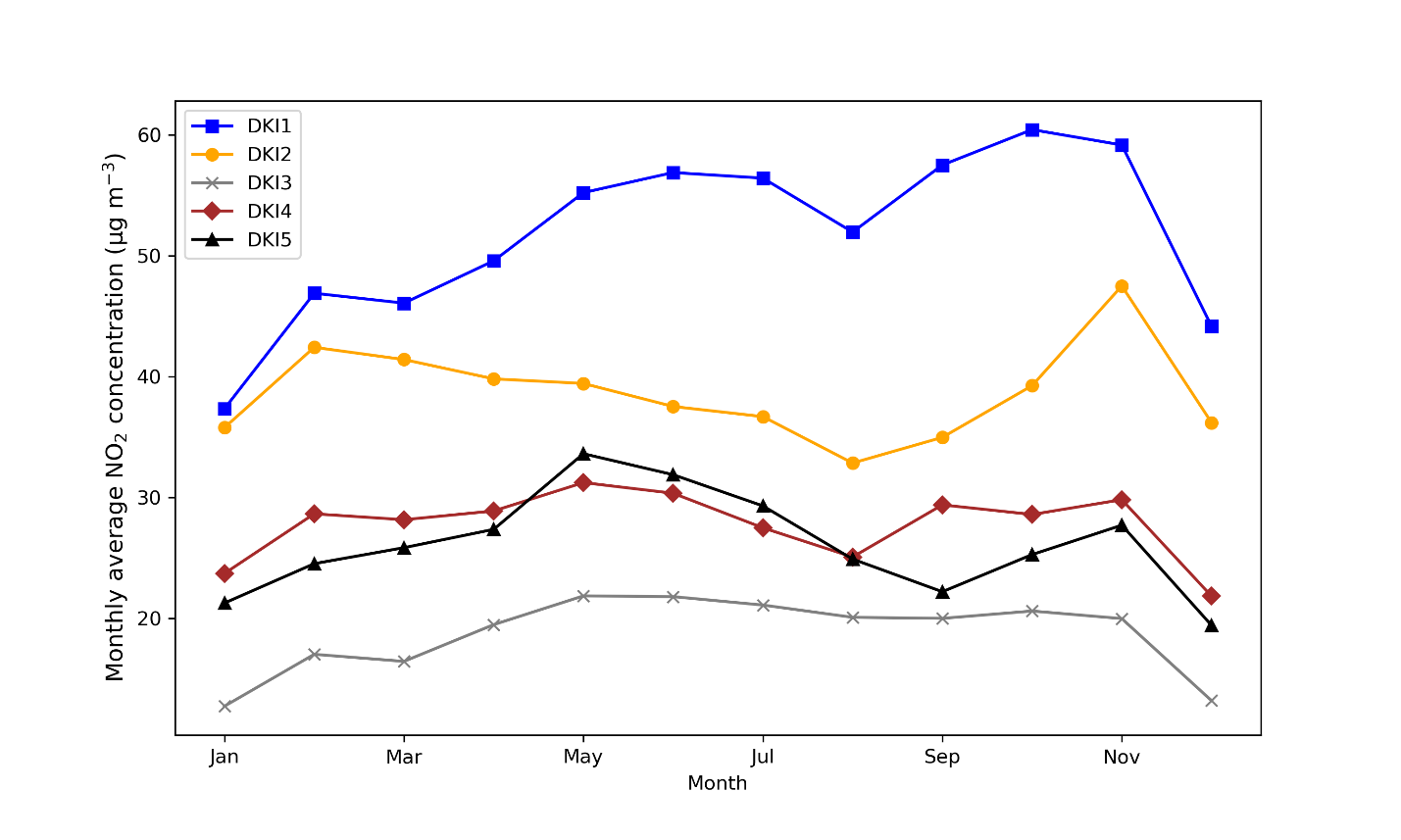


Figure S8. Seasonal variation of ground observed NO_2_ concentration at DKI sites from 2010 to 2019.


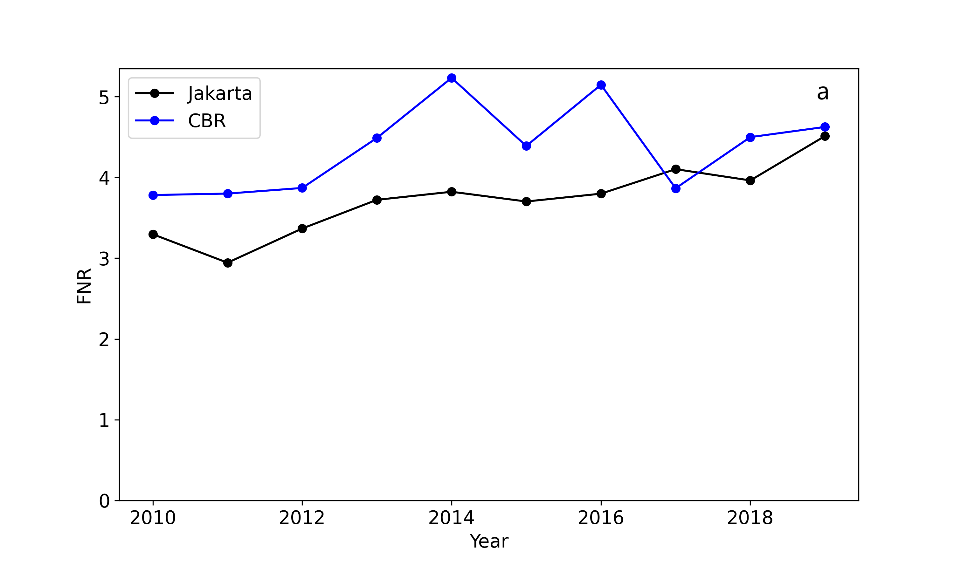


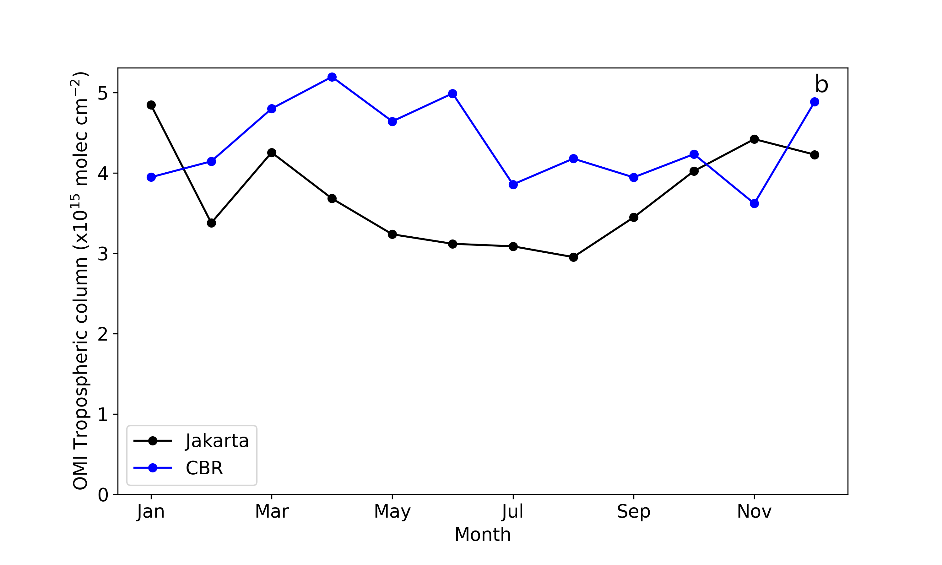


Figure S9. Time series of the (a) annual average and (b) seasonal variation in FNR in Jakarta and CBR from 2010 to 2019.

**
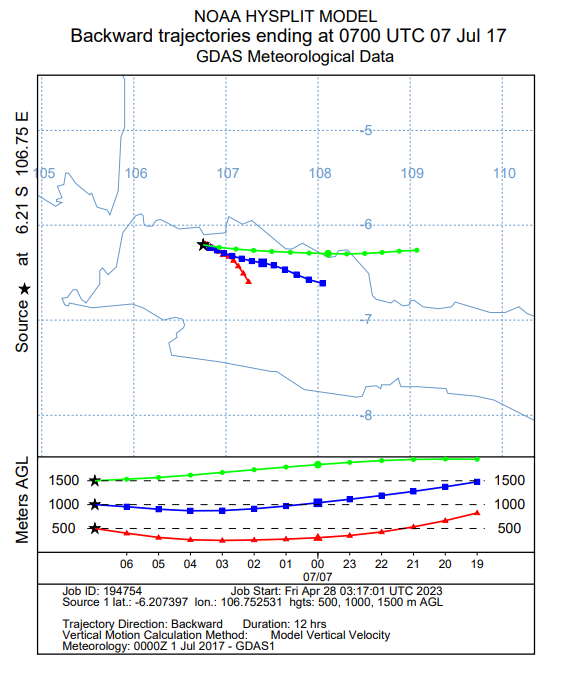
**

Figure S10. HYSPLIT backward trajectory on July 7, 2017 (dry period) in Jakarta.


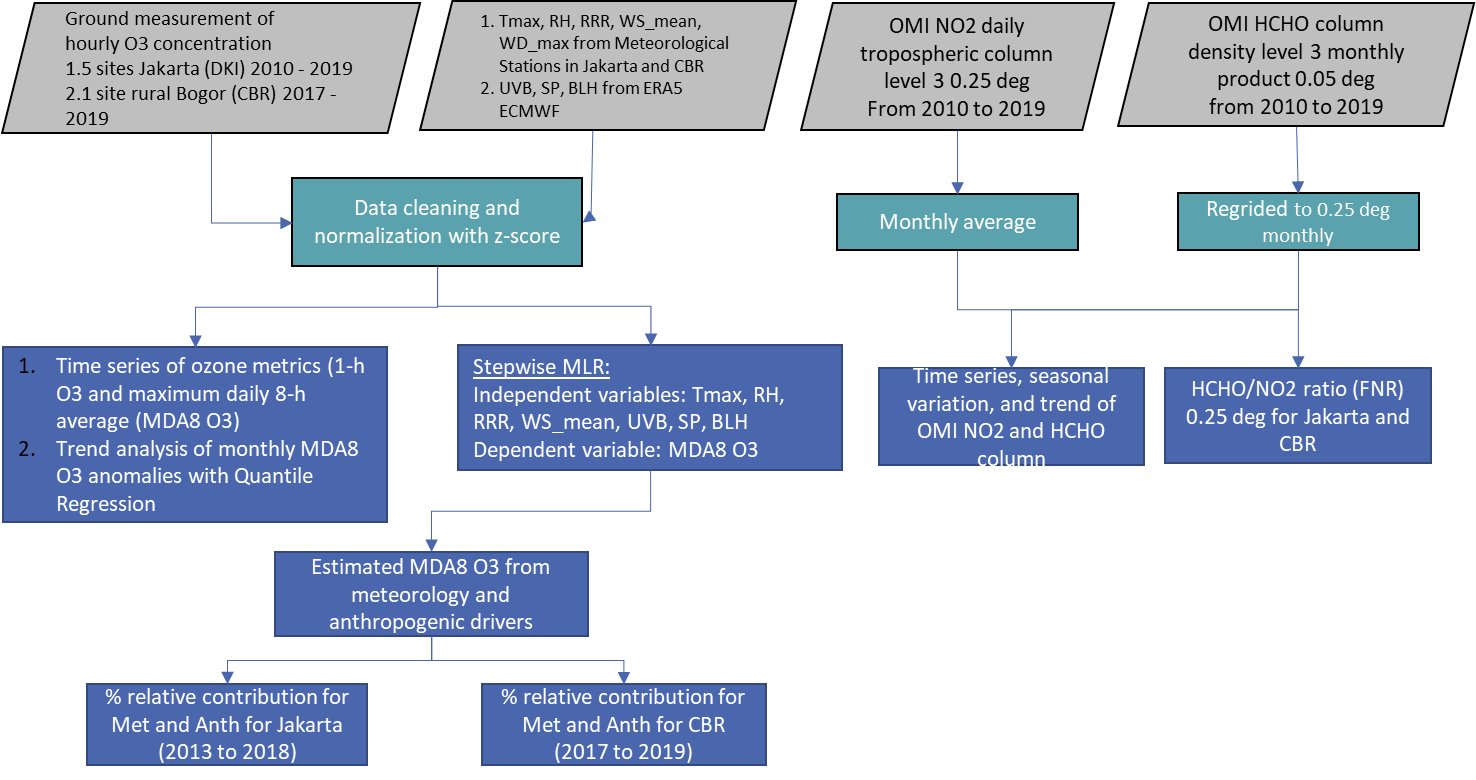


Figure S11. Flowchart and methodology used in this study.

**References**

1. Duncan, B. N. *et al.* Application of OMI observations to a space-based indicator of NOx and VOC controls on surface ozone formation. *Atmos. Environ.* **44**, 2213–2223 http://dx.doi.org/10.1016/j.atmosenv.2010.03.010 (2010).

2. Jin, X., Fiore, A., Boersma, K. ., De Smedt, I. & Valin, L. Inferring Changes in Summertime Surface Ozone−NOx−VOC Jin 2020.pdf. *Environ. Sci. Technol.* **54**, 6518–6529 (2020).

3. Wang, W., Ronald Van Der, R., Ding, J., Van Weele, M. & Cheng, T. Spatial and temporal changes of the ozone sensitivity in China based on satellite and ground-based observations. *Atmos. Chem. Phys.* **21**, 7253–7269 (2021).

4. Chen, Y. *et al.* Research on the ozone formation sensitivity indicator of four urban agglomerations of China using Ozone Monitoring Instrument (OMI) satellite data and ground-based measurements. *Sci. Total Environ.* **869**, 161679 (2023).

5. Santiago, J., Inoue, K. & Tonokura, K. Diagnosis of ozone formation sensitivity in the Mexico City Metropolitan Area using HCHO/NO2 column ratios from the ozone monitoring instrument. *Environ. Adv.* **6**, 100138 (2021).

6. Itahashi, S., Irie, H., Shimadera, H. & Chatani, S. Fifteen-Year Trends (2005–2019) in the Satellite-Derived Ozone-Sensitive Regime in East Asia: A Gradual Shift from VOC-Sensitive to NOx-Sensitive. *Remote Sens.* **14**, (2022).

7. National Statistical Agency. Population by Regency/City in DKI Jakarta Province (Jiwa), 2020-2022. https://jakarta.bps.go.id/indicator/12/1270/1/jumlah-penduduk-menurut-kabupaten-kota-di-provinsi- dki-jakarta-.html (2022).
